# Direct Visualization of Chemical Transport in Solid-State Chemical Reactions by Time-of-Flight Secondary Ion Mass Spectrometry

**DOI:** 10.1021/acs.nanolett.4c00021

**Published:** 2024-03-13

**Authors:** Sang T. Pham, Anh Kiet Tieu, Chao Sun, Shanhong Wan, Sean M. Collins

**Affiliations:** † Bragg Centre for Materials Research & School of Chemical and Process Engineering, University of Leeds, Woodhouse Lane, Leeds LS2 9JT, U.K.; ‡ School of Mechanical, Materials, Mechatronic and Biomedical Engineering, University of Wollongong, Wollongong, NSW 2522, Australia; § State Key Laboratory of Solid Lubrication, Lanzhou Institute of Chemical Physics, Chinese Academy of Sciences, Lanzhou 730000, P. R. China; ∥ School of Chemistry, University of Leeds, Woodhouse Lane, Leeds LS2 9JT, U.K.

**Keywords:** *in situ* heating, TOF-SIMS, ceramic coatings, hot corrosion, sodium diffusion

## Abstract

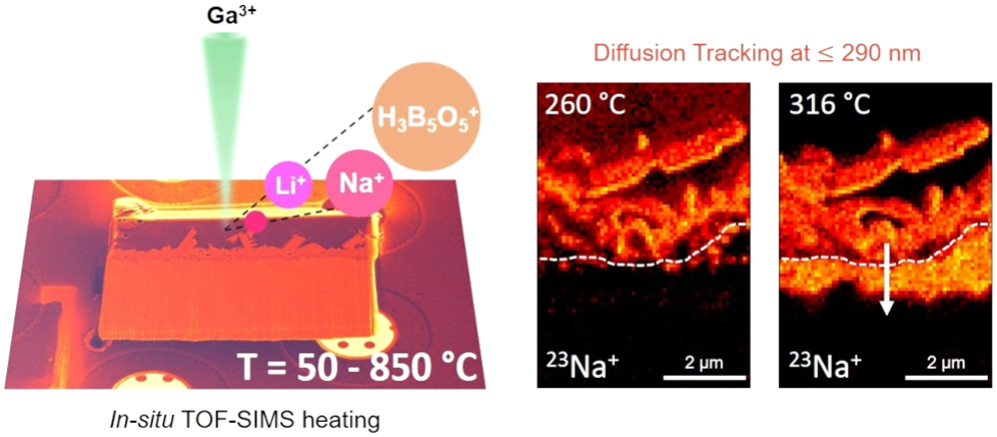

Systematic control and design of solid-state chemical reactions are required for modifying materials properties and in novel synthesis. Understanding chemical dynamics at the nanoscale is therefore essential to revealing the key reactive pathways. Herein, we combine focused ion beam–scanning electron microscopy (FIB-SEM) and time-of-flight secondary ion mass spectrometry (TOF-SIMS) to track the migration of sodium from a borate coating to the oxide scale during *in situ* hot corrosion testing. We map the changing distribution of chemical elements and compounds from 50 to 850 °C to reveal how sodium diffusion induces corrosion. The results are validated by *in situ* X-ray diffraction and post-mortem TOF-SIMS. We additionally retrieve the through-solid sodium diffusion rate by fitting measurements to a Fickian diffusion model. This study presents a step change in analyzing microscopic diffusion mechanics with high chemical sensitivity and selectivity, a widespread analytical challenge that underpins the defining rates and mechanisms of solid-state reactions.

Solid-state chemical reactions occur across multiple length scales, ranging from large-scale geological transformations within the Earth’s crust^1^ to the precise micro/nano-scale engineering of materials.^2^ A variety of intricate physical and chemical processes are pronounced at elevated temperature, including high-temperature oxidation and corrosion, temperature-induced mechanical stresses, elastic/plastic deformations, and phase transformations.^3−5^ A key driving force behind many of these processes is the enhanced chemical transport and accelerated kinetics of active elements and compounds across interfaces.^6^ Tracking temperature-dependent chemical dynamics at micro- to nanometer length scales in real time is paramount for providing crucial feedback to tailor the design of properties, performance, and stability of multimaterial structures,^1−3,7,8^ such as by identifying the temperature range at which desirable properties emerge or at which chemical products are formed and for understanding the underlying mechanisms of solid-state reactions.

Typical approaches to studying solid-state reactions, including post-mortem measurements of reaction products by X-ray diffraction (XRD)^9^ and nuclear magnetic resonance (NMR)^10^ or computational modeling,^11^ can identify the phases formed and their relative weights/fractions but miss important transient and minor phases. *In situ* heating experiments using transmission electron microscopy (TEM) combined with X-ray energy dispersive spectroscopy (EDS), diffraction, and energy filtered TEM (EFTEM) have been developed to map changes at the nanoscale,^12−20^ albeit with significant constraints on sample thickness and typically offering information restricted to composition, crystal structure, and morphology. Time-of-flight secondary ion mass spectrometry (TOF-SIMS) at nanometer resolution, made possible by the use of a gallium (Ga) focused ion beam (FIB), can address these challenges.^21^ The advantages of TOF-SIMS for imaging the distribution of elements and their bonding^22^ encoded in multiatom ions from the near-surface region of solids^23^ with trace detection (parts-per-million to parts-per-billion range^24^) and isotopic specificity make it a preferred choice for studying complex chemical processes in energy storage^25^ and hot corrosion.^26^ Developments in micro-electromechanical system (MEMS)-based heaters for *in situ* experiments in the FIB-SEM^27^ combined with the capability to prepare cross-sectioned specimens (see Figure S1 in the Supporting Information) allow for mapping temperature-dependent chemical dynamics across interfaces at much higher spatial resolution than in microtome-based SIMS. Here, we apply TOF-SIMS with *in situ* heating to track chemical transport (Figure S2) and phase formations during the hot corrosion reactions between sodium borate coatings and oxide scales on the surfaces of metals.

Inorganic glass coatings, comprising glass-forming compounds (e.g., P_2_O_5_, B_2_O_3_, and SiO_2_) and network modifiers (e.g., Li, Na, and K), are often used to protect steel, cast iron, or aluminum products from corrosion, oxidation, wear, and tear.^28,29^ In practice, they significantly benefit hot metal forming processes by forming viscous and lubricating melts that can protect the steel components from wear and oxidation.^29^ However, these melts can cause undesirable hot corrosion^26,30,31^ and form hard precipitated spinel oxides^32,33^ (Figure S3), leading to material losses and complicating descaling processes. Sodium has been strongly implicated in hot corrosion by these glass coatings,^26,30,34^ with its mobility hypothesized as the main factor.^30^ While *ab initio* calculations support the experimental hypothesis showing the diffusion of sodium from the borate glass to the Fe_2_O_3_ surfaces at elevated temperatures,^35,36^ no direct observation has elucidated this mechanism. We now seek to unambiguously identify the role of sodium in glass coatings below their transition temperatures by direct, real-time observations.

Figure 1a presents a simplified schematic of *in situ* TOF-SIMS heating experiments applied to a protective glass coating on the oxide layers at the surface of stainless steel. The sample comprises a sodium borate coating as the uppermost layer, followed by the intermediate oxide layers with the dendritic iron oxide grown above the continuous iron–chromium oxide layer. The cross-sectional sample was deposited on a MEMS heating chip (flat on the chip, perpendicular to the ion beam). TOF-SIMS analysis was performed using a Ga primary ion beam at an accelerating voltage of 30 kV and a current of 0.23 nA, scanned continuously across the sample surface throughout *in situ* heating. The lateral resolution of resulting images was estimated as better than 290 nm at an abrupt step in the ^23^Na^+^ signal (20%–80% of the maximum intensity; see Figures S4–S6), a conservative estimate as it assumes a sharp, edge-on interface. Here, isochronal heating was applied from 50 to 850 °C (1 °C/s), producing a *Z*-direction (direction of successive two-dimensional scans) that directly tracks the temperature. Each frame’s acquisition time was 1.4 s, allowing for real-time observation of chemical dynamics. A list of peaks found in the mass spectrum (a “peak list”) was generated at every probe position, resulting in a hyperspectral data cube at each temperature that contains information about both chemical elements and compounds.

**Figure 1:**
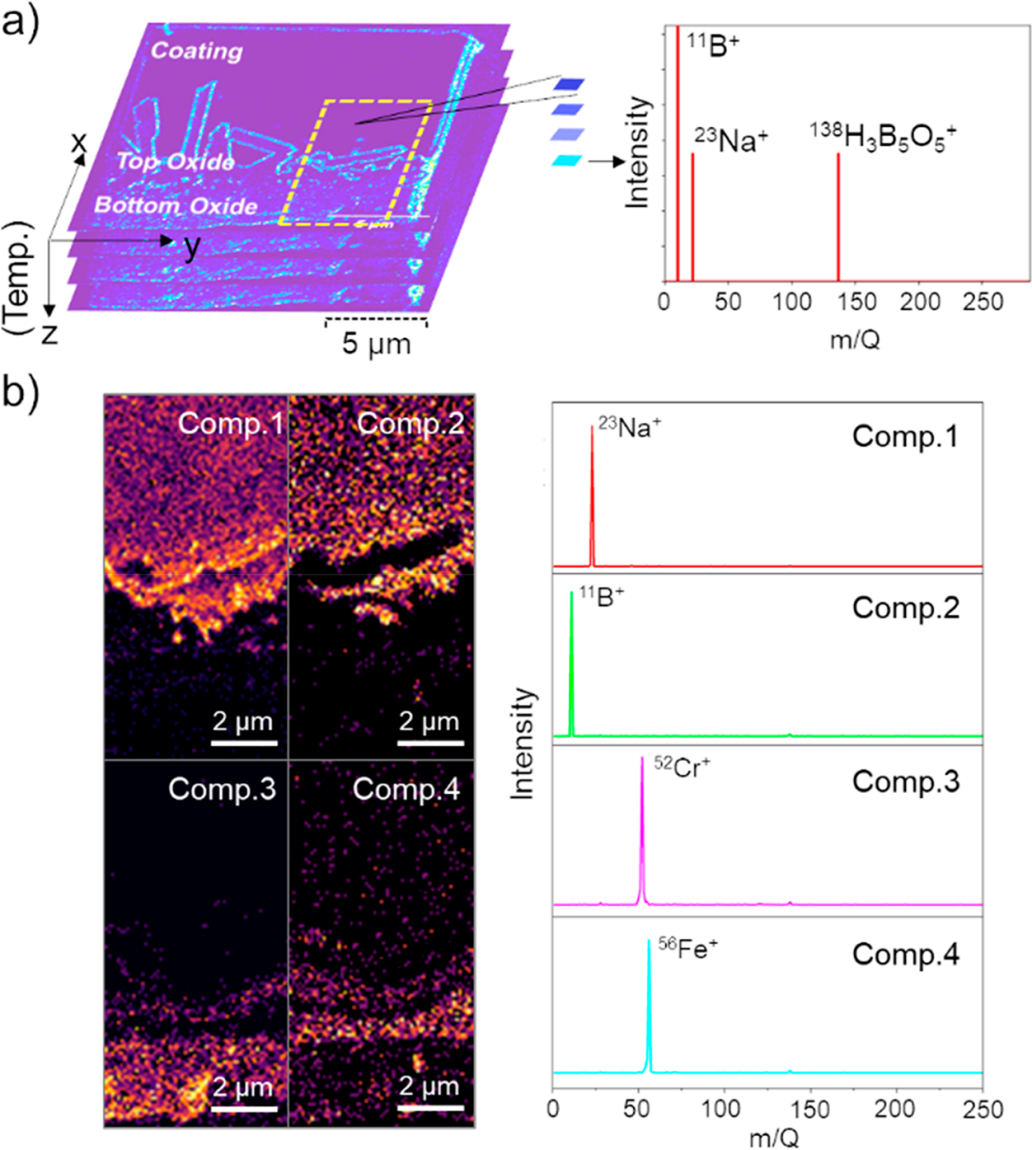
Correlated elemental and compound maps within a sodium borate coating and oxides cross sections, enabled by TOF-SIMS experiments and multivariate analysis. (a) Schematic illustration of spatially resolved and high-temperature resolution (1.4 s/frame at 1 °C/s, resulting in 1.4 °C/frame) data set from *in situ* TOF-SIMS heating experiments. (b) Four distinct components (Comp. 1–4) from sodium borate and oxides, extracted by NMF analysis, directly mapping the spatial distribution of ^11^B^+^, ^23^Na^+^, ^52^Cr^+^, and ^56^Fe^+^ within the cropped area marked by the yellow dashed line in (a).

Figure 1b shows four spatial maps and their associated spectral factors at a temperature of 250 °C, extracted through non-negative matrix factorization (NMF). The region of interest is cropped from the larger area used for data collection (yellow dashed line in Figure 1a), offering a simpler, multilayered structure for close analysis. NMF, a blind source separation technique rooted in machine learning, can effectively disentangle multiple contributing signals (sources) within a spectrum image,^37−39^ decomposing the data set into a few spectral factors and corresponding maps while excluding factors that describe noise. The number of components used in NMF was determined using established procedures^37,39^ (see the Supporting Information and Figure S7). In this instance, we found that eight non-negative components adequately described the data set. In Figure 1, we show only the four main components with spectral factors primarily presenting ^11^B^+^, ^23^Na^+^, ^52^Cr^+^, and ^56^Fe^+^ peaks. The next four components show the peaks for ^10^B^+^, ^51^Cr^+^, ^53^Cr^+^, and ^55^ Mn^+^ (Figure S8).

Figure 2 shows NMF component maps representing the distribution of ^11^B^+^, ^23^Na^+^, ^52^Cr^+^, and ^56^Fe^+^ ions within the same area at 250, 350, and 420 °C. Figures S8–S10 show the corresponding maps (when observed) for ^22^Na^+^, ^10^B^+^, ^51^Cr^+^, ^53^Cr^+^, ^55^ Mn^+^, and ^138^H_3_B_5_O_5_^+^, and Figure S11 further shows the consistent temperature-dependent spatial distributions observed for the isotopes of Na^+^, B^+^, Cr^+^, and Fe^+^. At 250 °C (Figure 2a), ^11^B^+^ and ^23^Na^+^ coexisted primarily in the uppermost coating layer, with some infiltration of ^23^Na^+^ into interfacial areas between the coating and oxide layers. This infiltration of sodium could result from reactions between the coating and the oxide layers during sample preparation, where rapid soaking of the samples at 600 °C was conducted, forming the reaction products of sodium and iron oxides.^26,40^ At this temperature, NMF maps indicate a distinct separation of the iron oxide and chromium oxide layers. Additionally, the NMF map for component 4 reveals a spatial distribution of ^56^Fe^+^ within the sodium borate coating, suggesting the partial dissolution of iron oxide particles as a consequence of sodium infiltration in some dendrite oxide areas.

**Figure 2:**
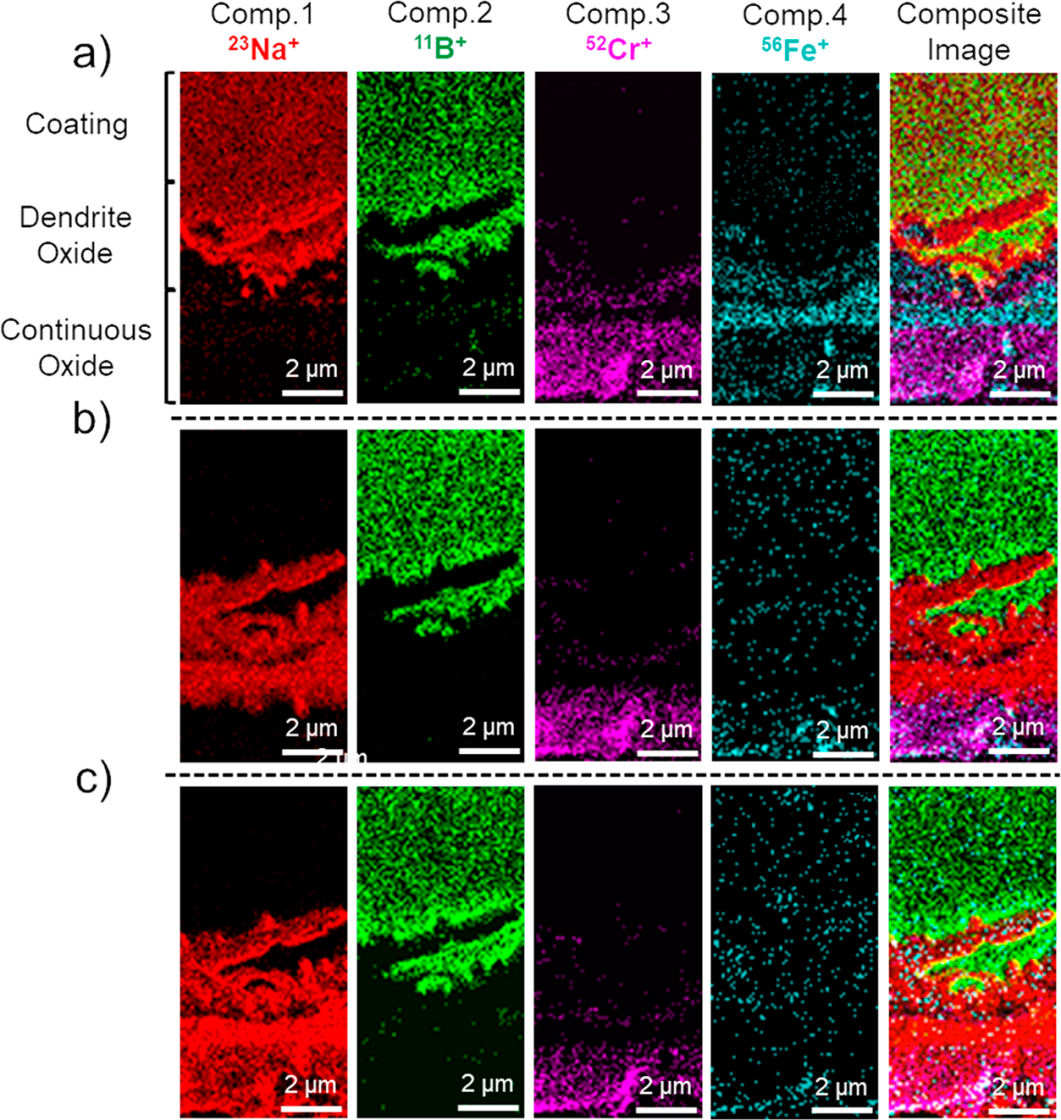
Diffusion of sodium from the borate coating to the oxide layers during isochronal heating is visualized by multivariate analysis of TOF-SIMS results at cropped individual temperatures. NMF maps of four selected distinct components, representing the distribution of ^11^B^+^, ^23^Na^+^, ^52^Cr^+^, and ^56^Fe^+^ within the selected field of view, are at (a) 250, (b) 350, and (c) 420 **°**C. The maps at 250 **°**C are repeated from Figure 1b for clarity of comparison.

As the temperature increased to 350 °C (Figure 2b), a distinct spatial separation emerged between ^11^B^+^ and ^23^Na^+^. While ^23^Na^+^ was observed within the iron oxide regions, ^11^B^+^ remained concentrated in the coating. Simultaneously, the NMF map for component 4 showed a more intense distribution of ^56^Fe^+^ ions within the borate coating, accompanied by a significant decrease in intensity in the areas that were originally iron oxide. Notably, the spatial distribution of ^52^Cr^+^ remained consistent under heating with no signal contribution of these ions in the borate coating. At 420 °C, when sodium largely infiltrated the oxide layers (Figure 2c), the NMF maps revealed enhanced signals of ^56^Fe^+^ in the borate coating. The distribution of ^52^Cr^+^ within the map remained confined to the areas surrounding the chromium oxide layer. Iron present in the borate coating likely originates from the iron oxide layers, implying that portions of these areas were dissolved. Meanwhile, the absence of ^52^Cr^+^ signals in the borate coating signifies no or a very low level of chromium oxide dissolution at these temperatures.

We previously observed diffusion of sodium from the borate glass to the oxide scale by post-mortem TEM-EDS^26,32^ in experiments at or above the glass transition temperature of borate glasses (∼520 °C under vacuum conditions, Figure S3b). Here, we show that sodium diffusion and the partial dissolution of the iron oxide layer occurs within a much lower temperature range (260–420 °C, Video S1), providing crucial validation for indirect observations and calculations of sodium diffusion in borate glasses at temperatures below their glass transitions.^41,42^ We have ruled out ion scanning induced chemical transformations^43−45^ using control experiments by TOF-SIMS and *in situ* XRD (see Figures S12–S14), in line with previous comparisons between post-mortem TEM-EDS and *in situ* TOF-SIMS analyses in other material systems.^46^ These observations also confirm that the *in situ* TOF-SIMS observations from the first few nanometers of the surface reflect processes in the bulk. The concomitant changes observed, with each technique capturing distinct information, indicate a common origin but also prompt further work to tackle challenging multitechnique integration.

This diffusion during early stages of isochronal heating and its role in the partial dissolution of iron oxide layers are likely driven by the creation of new chemical phases. To interrogate this hypothesis, we focus on selected mass per charge number (*m*/*Q*) regions of mass spectra for the borate coating and the two oxide layers (Figure 3). These spectra were acquired within the temperature range 200–510 °C, capturing information about the dynamic formation of chemical compounds before reaching the glass transition temperature of sodium borate (∼520 °C in a vacuum, Figure S3). The borate coating showed clear signs of iron incorporation with the detection of ^56^Fe^+^ above 200 °C. The intensity of this secondary ion gradually increased with temperature (Figure 3a). Meanwhile, a sharp decrease in the intensity of ^23^Na^+^ occurred above 340 °C (Figure S15), aligning with the observed sodium diffusion (Figure 2). The mass spectra also revealed the presence of iron borate by the detection of ^100^FeHBO_2_^+^ as well as other secondary ion fragments reflecting the borate glasses, such as ^31^H_4_BO^+^, ^119^B_5_O_4_^+^, and ^138^H_3_B_5_O_5_^+^, above 400 °C (Figure 3a,b). In the iron oxide layer, the mass spectra showed the detection of ^79^NaFe^+^ at ∼200 °C, and its intensity increased with temperature, indicating the gradual formation of NaFeO_2_ phases before sodium borate melted (Figure 3c). Similarly, in the chromium-rich layer, the mass spectra included ^75^NaCr^+^ above 342 °C, suggesting the formation of NaCrO_2_ phases. These observations align with prior studies reporting the formation of NaFeO_2_ and NaCrO_2_ phases at relatively low temperatures.^41^

**Figure 3:**
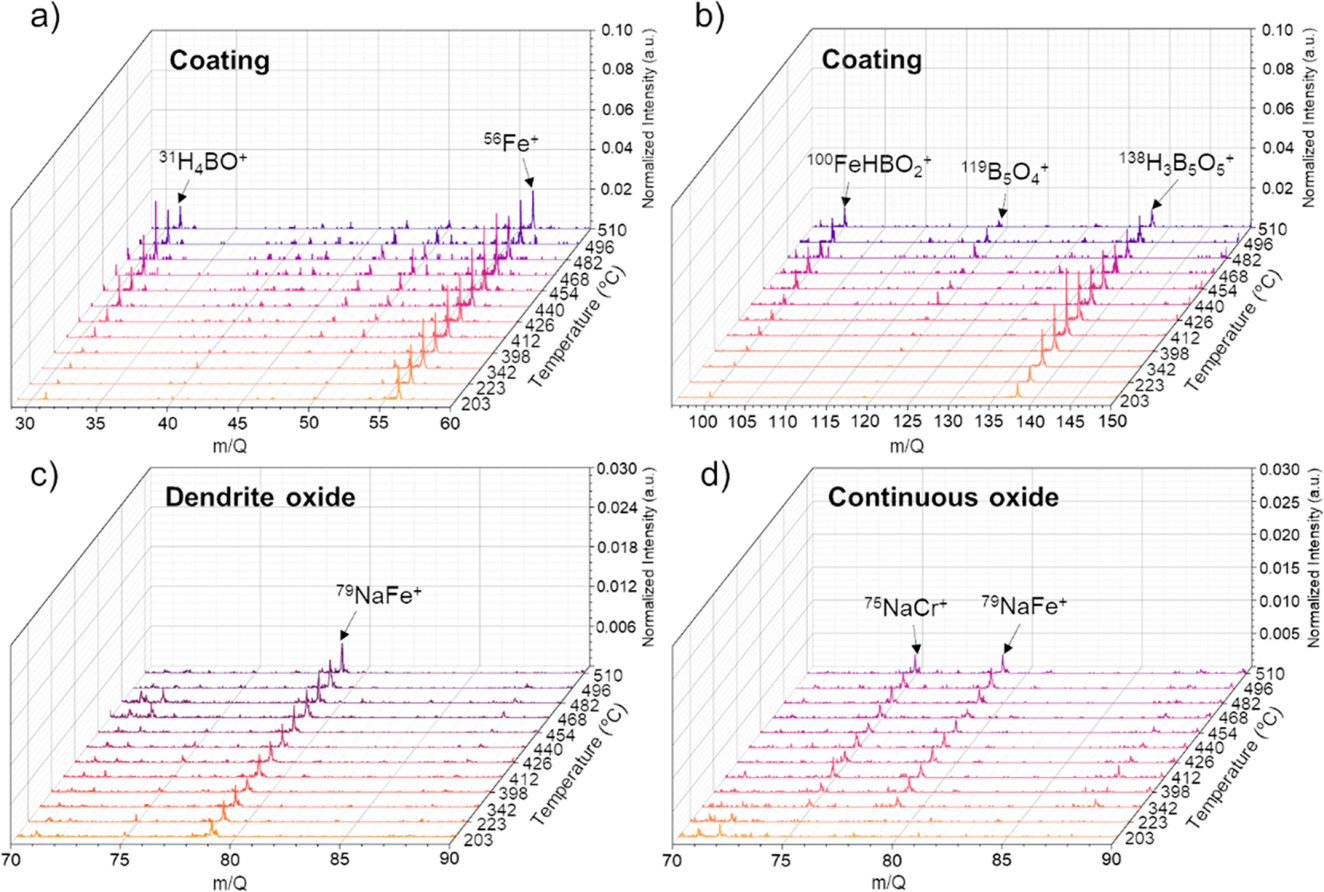
Tracking chemical phase formation in the borate coating and oxide layers during isochronal heating. (a, b) Mass spectra at different temperatures, extracted from the sodium borate coating layer showing separated *m*/*Q* ranges for better visualization. (c) Mass spectra at different temperatures, extracted from the iron oxide layer. (d) Mass spectra at different temperatures, extracted from the chromium oxide layer.

The formation of these two phases (NaFeO_2_ and NaCrO_2_) indicates that the occurrence of the basic dissolution reactions between iron oxides, chromium oxides, and sodium borate,^47^ proposed previously to occur between the melts and solid oxides. Below the melting temperature, we hypothesize that the basic dissolution reactions follow electrochemical pathways:
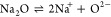
1
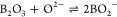
2
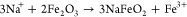
3
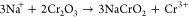
4In sodium borate fused salts, Na_2_O and B_2_O_3_ phases coexist. The basicity of the fused salt is defined by the Na_2_O activity which corresponds to the activity of O^2–^ because Na_2_O can be decomposed into Na^+^ and O^2–^ at high temperatures (eq 1). The formed O^2–^ can then ionize part of the B_2_O_3_ network into the BO_2_^–^ ions (eq 2) when Na^+^ is generated.^48^ The reverse transformations of Na_2_O and B_2_O_3_ (eqs 1 and 2) are expected to remain at the thermodynamic equilibrium.^48,49^ However, as the temperature rises sufficiently for reactions 3 and 4 to occur, diffusion of Na^+^ from the coating toward the oxide layers occurs. This diffusion shifts reaction 1 to the right, generating more Na^+^ and BO_2_^–^ in the coating network. Electrochemical reactions between Na^+^ and oxides (eqs 3 and 4) result in the gradual formation of NaFeO_2_ and NaCrO_2_ within the oxide layers, as detected in the TOF-SIMS mass spectra (Figure 3c,d). Complementary *in situ* XRD heating (Figure S14) verified the formation of NaFeO_2_ phase at the same temperature range.

As Na^+^ ions depart from the glass, the remaining BO_2_^–^ ions result in a negatively charged boron oxide network. To balance the charge of this boron oxide network, metallic cations formed in reactions 3 and 4 migrate to the borate coating. Previous studies suggest that metallic ions, including M^2+^ and M^3+^ cations (M = Fe, Mn, Cr), can integrate into the glass network to form the inorganic glass matrix.^49^ However, as indicated by NMF analysis (Figure 2), iron cations predominantly fulfill this role, causing selective dissolution of iron oxide over chromium oxide. Similar observations were reported previously,^47^ attributable to the smaller ionic radius of Fe^3+^ compared to Cr^3+^ ions^50^ with the harder Fe^3+^ ion interacting more favorably with hard bases (BO_2_^–^ in this case), an interpretation supported by the detection of the secondary ion fragments of iron borate compounds (Figure 3b).

Further examination of the mass spectra from the two oxide layers (Figures S16 and S17) revealed an increase in the intensity of a peak assigned to ^7^Li^+^, suggesting the incorporation of lithium within the oxide layers at high temperatures. Lithium may originate from trace contaminants in the sodium borate, reported to have a purity of 99.5 wt %. Examination of the mass spectrum exported from the coating at low *m*/*Q* (Figure S15c) suggests the presence of ^7^Li^+^ below 223 °C. As the temperature increased above 223 °C, the ^7^Li^+^ peak disappeared in the coating region, while it gradually appeared in the oxide layers (Figures S16 and S17), becoming detectable above 500 °C and increasing with temperature (Figures S15 and S16). The ^7^Li^+^ intensity grew significantly when the temperature rose above the transition point (520 °C), i.e., 580 °C (Figure S16d), suggesting lithium diffusion from the coating to the oxide scale although we cannot rule out increased detection due to temperature-dependent ion yields. NMF analysis, performed on the TOF-SIMS data set cropped at 580 °C (Figure S18), depicts the spatial distribution of lithium localized within the oxide layers. Lithium may diffuse to and react with the oxide layers in a fashion similar to that of sodium. The detection of lithium at trace levels in this study highlights the substantial potential of TOF-SIMS analysis for investigating the impact of impurities on solid-state reactions.

The visualization of the chemical propagation fronts by *in situ* FIB-SIMS, capturing propagation from within a receding sputtered surface, also supports direct measurement of mass transfer rates. The diffusion coefficient of active elements in solid-state materials have been measured more commonly using radiotracer methods combined with grinder sectioning,^42^ differential calorimetry,^51^*in situ* TEM heating,^52^ and calculations.^53^ Here, using *in situ* FIB-SIMS mapping, we can directly track the motion of sodium ions in the oxide layer to extract a temperature-dependent diffusion coefficient of sodium. Figure 4a provides an overview image of the specimen generated by integrating the ^23^Na^+^ intensity from 200 to 315 °C, illustrating the spread of sodium from the borate coating to the oxide layers. Cyan boxes mark the areas used for the measurement of the transformed segment length, *L*.

**Figure 4:**
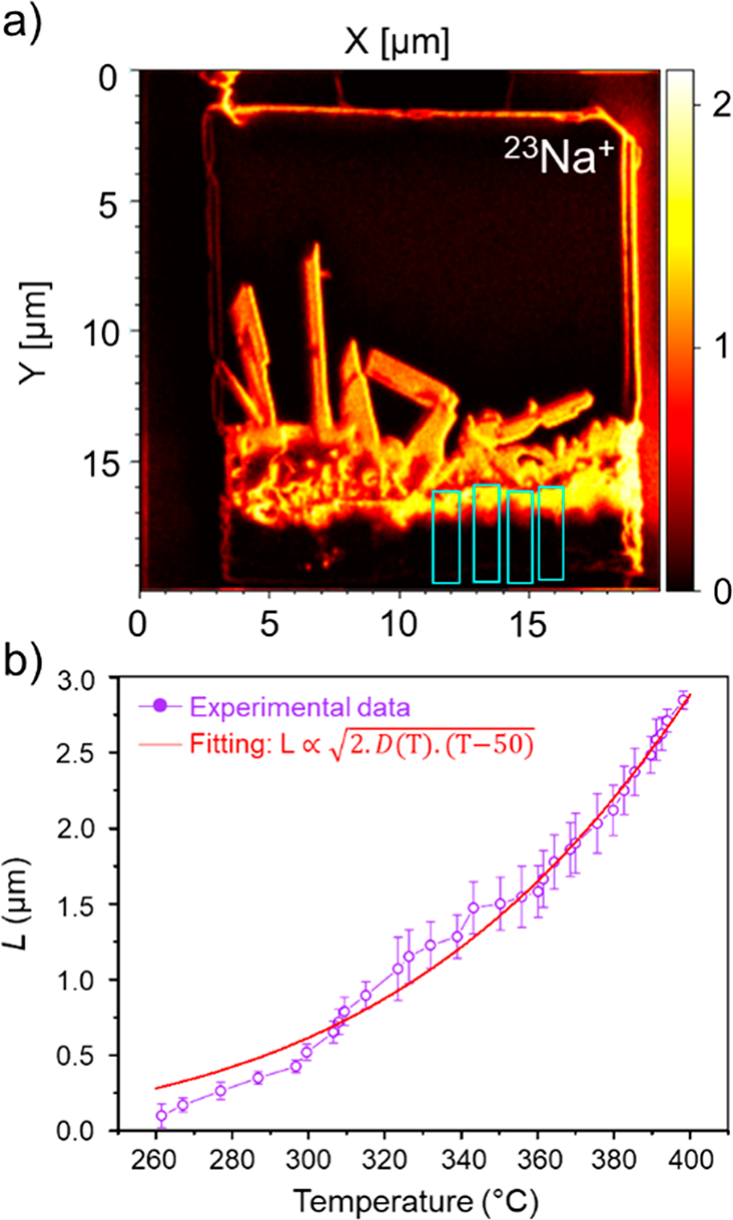
Quantitative analysis of chemical dynamics, enabled by direct tracking of sodium diffusion during heating. (a) An ion image showing a map of ^23^Na^+^ within the cross-sectioned specimen, constructed by summing intensity signals from 200 to 315 °C. The inset cyan boxes mark the areas which are selected to measure the diffusion length of sodium via temperature. (b) Diffusion length (*L*) versus temperature in the homogeneous oxide layer, averaged from the measurements at four different areas. The error bars show one standard deviation estimated from the four measurements of *L* at each temperature. The red solid line shows the curve fitting to experimental results using eq i.

For simplicity, we used the temperature range of 260–400 °C for this analysis (corresponding to ∼30–40 nm removed by sputtering; see the Supporting Information), as the motion of sodium exhibited a unidirectional propagation from the top to the bottom of the continuous oxide layer in this range. In contrast, sodium diffused in various directions within the loosely packed dendritic oxide layer, complicating the measurement of the sodium diffusion length. Measurements of *L* were performed by drawing a line from the start of the box (*x*_0_), i.e., the boundary between continuous oxide and loosely packed oxide, to the maximum vertical extent of the sodium front (*x*_i_) (see the Supporting Information and Figure S2). Minor deviations from a perfectly flat interface within and near the box may under- or overestimate *L* for convex or concave interfaces, respectively, contributing to measurement uncertainty. We accordingly averaged the measurements in four areas (Figure 4a). We also assume that such unidirectional motion of sodium in the continuous oxide layer was driven by concentration (a diffusion process) rather than under the influence of the potential. The measured *L* showed a nonlinear increase with temperature (Figure 4b). Building on Fick’s second law of diffusion and a time-dependent diffusivity model, we derived a solid-state diffusion model for sodium within the continuous oxide layer (see the Supporting Information):
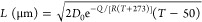
iwhere *D*_0_ (μm^2^ s^–1^) is a pre-exponential factor, representing the diffusion coefficient at infinite temperature, *Q* (J mol^–1^) is an activation energy for the diffusion, *R* is the gas constant (8.314 J mol^–1^ K^–1^), and *T* (°C) is the temperature. In eq i, *D*_0_ and *Q* are constant and serve as fitting parameters.

Figure 4b shows a fit to eq i overlaid on the experimental data, demonstrating correspondence between the experimental data and the functional form of the diffusion model. Analysis of the goodness-of-fit affirmed the model effectively explains the experimental data (Supporting Information and Figure S19) and showed no indications of additional interactions between surface diffusion and sputtering. *D*_0_ and *Q* were estimated by fitting (Table S1), enabling us to tabulate the diffusion coefficient (*D*(*T*)) of sodium in the oxide layers (Table S2) using an Arrhenius-type relationship (eq S7). The calculated diffusion coefficient of sodium in the oxide layer falls within the range of 2.0 × 10^–12^–1.2 × 10^–10^ cm^2^/s for the temperature range 260–400 °C, in agreement with sodium diffusivity calculated in solid oxides, 9.2 × 10^–12^–5.9 × 10^–8^ cm^2^/s using density functional theory.^53^

In summary, we have revealed that partial dissolution of the oxide scale can occur at temperatures below the glass transitions of sodium-containing inorganic glass coatings. This dissolution process is instigated by diffusion of sodium from the coatings to the oxide scale. The capacity to obtain temperature profiles for the diffusion length of active elements, i.e., sodium, by *in situ* TOF-SIMS, enables us to quantify the diffusion coefficient. This information serves as a valuable input for computational models of the chemical dynamics occurring in the hot corrosion processes of other inorganic glasses with complex compositions, offering insights for mitigating the corrosion reactivity at high temperatures. More widely, *in situ* heating of TOF-SIMS opens the exploration of chemical dynamics at high temperature in applications from metal-forming and engine lubricants to nuclear reactor components to advance the understanding of performance degradation through to new materials synthesis routes.

## Data Availability

The data underlying this study are openly available in the University of Leeds Data Repository at https://doi.org/10.5518/1495.

## Supporting Information Available

Experimental details; *in situ* SEM heating results; lateral resolution determination; evaluation of beam conditions for TOF-SIMS analysis; demonstration of multivariate data analysis and results for the TOF-SIMS data set at 250, 350, 420, and 580 °C; note on effect of ion beam irradiation on chemical transport; *in situ* XRD heating results, the mass spectra extracted from the coating; the loosely packed oxide layer; and the continuous oxide layer for individual temperatures; note on established Fickian diffusion model and evaluation of the goodness-of-fit for the model; diffusion coefficient exported for individual temperatures (PDF)
Video S1: diffusion of sodium from the coating to the oxide scale at 260–400 °C (AVI)

## Supplementary Material

nl4c00021_si_001.pdf

nl4c00021_si_002.avi
