# Supplementary material for: Direct Visualization of Chemical Transport in Solid-State Chemical Reactions by Time-of-Flight Secondary Ion Mass Spectrometry
Source: Nano Lett. 2024 Mar 13;24(12):3702–9. doi: 10.1021/acs.nanolett.4c00021 (PMC10979428; doi:10.1021/acs.nanolett.4c00021)
Supplement: Supplementary file 1 — nl4c00021_si_001.pdf [file nl4c00021_si_001.pdf]

Supplementary Information

Direct visualization of chemical transport in solid-state chemical reactions by time-of-flight secondary ion mass spectrometry

*Sang T. Pham[a]**, *Anh Kiet Tieu[b]*, *Chao Sun[a]*, *Shanhong Wan[c]*, *and Sean M. Collins[a,d]**

[a]Bragg Centre for Materials Research & School of Chemical and Process Engineering, University of Leeds, Woodhouse Lane, Leeds LS2 9JT, UK

[b]School of Mechanical, Materials, Mechatronic and Biomedical Engineering, University of Wollongong, Wollongong NSW 2522, Australia

[c]State Key Laboratory of Solid Lubrication, Lanzhou Institute of Chemical Physics, Chinese Academy of Sciences, Lanzhou, 730000, PR China

[d]School of Chemistry, University of Leeds, Woodhouse Lane, Leeds LS2 9JT, UK

AUTHOR INFORMATION

Corresponding Author

***Sang T. Pham, T.S.Pham@leeds.ac.uk

***Sean M. Collins, S.M.Collins@leeds.ac.uk

Experimental Section

Sample preparation. The coatings of glass-like sodium borate ($Na_2B_4O_7$) on oxide scales of stainless steel were prepared using the process reported before[1, 2], using sodium tetraborate decahydrate ($Na_2B_4O_7.10H_2O$) purchased from Merck (CAS: 1303-96-4, purity: ≥99.5%). Briefly, the process involved depositing the 6 wt % sodium borate aqueous solution onto a stainless steel disc (0.15 mL.cm^{-2}), followed by mild heating at 100 °C for 10 minutes before rapid annealing at 600 °C for 10 seconds. The cross-section of the coated steels from the area of interest were exposed by conducting ion milling inside a dual-beam FIB-SEM (FEI Helios NanoLab G3 CX) followed by lift-out using a micromanipulator (Omniprobe). The bulk material removal was conducted at 30 kV voltage and 9 nA probe current and the current was dropped to 2 nA for cleaning the cross-section. Before the lift-out step, the stage holder was rotated by 90°. The lift-out process was conducted under the geometry presented in Figure S1a. The coarse cross-section, mounted on the tungsten tip, was then cleaned further with the lamella hanging in space using an ion beam voltage of 5 kV and an ion beam current of 16 pA to obtain a clear hierarchical structure between the borate coating and oxide scale. Subsequently, the tungsten tip was rotated by 180° to align the cross-section specimen parallel to the MEMS-heating chip surface. The cross-section specimen was then gently deposited on the chip surface and fixed by platinum welding (Fig. S1b).

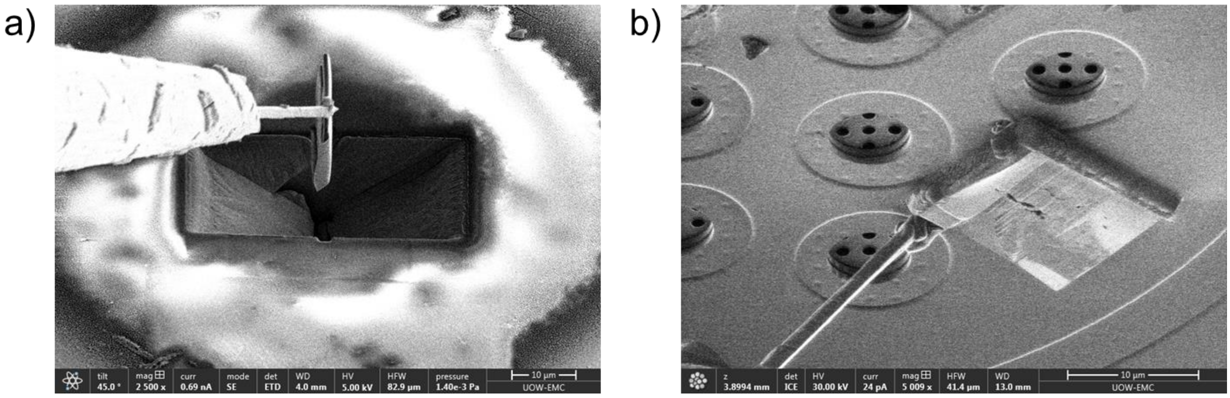

Figure S1. The geometry of the lift-out process for sample preparation on the MEMS heating chip. (a) Electron image showing the lift-out process being conducted at 90° compared to the standard procedure. (b) Electron image showing the rotation of the tungsten tip by 180° to align the specimen parallel with the MEMS heating chip followed by the deposition of the specimen on the chip surface by platinum welding.

FIB milling can modify samples, and so we considered these effects in our approach. One major consideration is the effect of any redeposition or modified surface layers following ion bombardment. Redeposition occurs as species sputtered by the ion beam are not completely evacuated by the vacuum system and reattach elsewhere on the surface. Surface layers can also exhibit implantation and structural changes. Generally, redeposition occurs primarily at the coarse milling step when removing large quantities of material. We have performed two internal cleaning steps after the bulk material removal (30 kV, 9 nA): (i) before the lift-out (30 kV, 2 nA); and (2) after the lift-out and before the deposition of the specimens on the heating chip (5 kV, 16 pA). The cleaning steps were

performed at lower voltage and current to achieve higher milling precision and slower milling rate to minimize the sample damage and material redeposition. We have conducted non-negative matrix factorization (NMF) analysis to evaluate the spatial distribution of the chemical elements (Figure S5b) in the sample before the heating experiments. We observed negligible intermixing of the materials between the coating and the oxide layers, indicating that the FIB sample preparations introduced minimal artefacts (e.g., new material from redeposition) or other redistribution of species within the samples.

***In situ* heating SEM imaging.** *In situ* heating SEM imaging was carried out in the dual-beam FIB-SEM vacuum chamber (FEI Helios NanoLab G3 CX) using a Thermo Scientific™ µHeater holder[3, 4]. Following the deposition of the specimen on the MEMS-based heater, heating was commenced from 200 °C to 1000 °C (heating rate of 1 °C/second) while SEM imaging was conducted simultaneously during the heating process. Imaging conditions were set at an electron beam voltage of 5 kV and an electron beam current of 1.4 nA, with a working distance of 6.8 mm and a horizontal full width of 41.4 µm. These settings resulted in an acquisition time of 0.2 seconds per image which was more than sufficient for capturing any structural changes in the borate coating and oxide layers in real time. To enable a qualitative assessment of chemical transport through SEM imaging, backscattered electrons (BSE) were used for image construction. In BSE imaging, the detected electron signal includes contributions from deeper locations within the specimen, albeit at lower resolution compared to secondary electron images. BSE can also provide analytical data regarding average elemental distributions, though not elemental identities, as the BSE signal intensity, i.e. image contrast, is strongly related to the atomic number (Z) of the elements present within the specimens.

***In situ* heating TOF-SIMS experiments.** TOF-SIMS analyses were conducted on the specimens during isochronal heating using the same µHeater holder[3] and in the FEI Helios NanoLab G3 CX. The TOF-SIMS analyzer (fibTOF), provided by TOFWERK, is integrated into the FIB-SEM (FEI Helios NanoLab G3 CX) at a dedicated port. The mass spectrometer uses an orthogonal design with pulsed secondary high voltage. The secondary ions are collected in pulser and pulsed in orthogonal direction to the detector. This configuration of the analyzer allows for continuous sputtering of the samples by the FIB ion beams. The collection optics are 8 mm from the sample, and the analyzed mass range at the current setup is 6-300 m/Q with the mass resolution estimated at 1150M/ΔM for $^{115}In^{+}$. The lateral resolution, dependent on the FIB performance and operating conditions, can achieve up to 47 nm. The detection limit is ~10ppm, determined for $^{6}Li^{+}$ in quartz. The pressures are $<9 \times 10^{-5}$ Pa in analysis chamber and 10^{-6} Pa in the TOF chamber, which has a separate ion pump. Both positive and negative secondary ions can be detected with this instrument but this study will concentrate on positive secondary ions.

For experiments with cross-sectioned specimens, the chip was heated to 50 °C directly prior to the *in situ* heating TOF-SIMS analysis. The dynamic heating was conducted from 50 to 850 °C with a heating rate of 1 °C/second. During the heating period, the TOF-SIMS analysis was performed

dynamically using an ion beam voltage of 30 kV and an ion beam current of 230 pA with a horizontal full width of 20 µm that resulted in the acquisition time for each frame of 1.4 second. These sputtering conditions were chosen by evaluating the trade-off between signal-to-noise ratio, mass resolution, and intensity ratio between interested ions and gallium ions (see below, Fig. S5-S6). Based on sputtering yields estimated for 30 kV Ga ion beams as approximately 0.3 μm^3/nC as established previously for a range of materials including metals, semiconductors, and oxides (alumina) at room temperature[5, 6] and accounting for the 30 kV Ga ion beam current, exposure time, and area scanned, we estimate a sputtering rate of approximately 0.2 nm/s, though elevated temperature may further modify sputtering rates. The flight time was set at 12 µs per pixel resulting in a mass range of 0-288 m/z. An initial group delay of 1500 ns before detection was set to eliminate ringing artefacts which prevents detection of ion species below 5 m/z. During TOF-SIMS analysis, an electron flood gun was constantly applied on the specimen to neutralize the charging effect caused by the non-conductive sodium borate coating. Low energy electrons (2 kV) with an electron beam current of 11 nA current from the SEM column were used to irradiate the sample, oriented at an angle of 52° to the sample's plane normal with a working distance of 10 mm (over-focus). Controlled TOF-SIMS analysis at individual temperatures was performed under the same conditions using an ion beam voltage of 5 kV and an ion beam current of 68 pA to evaluate the reliability of the results (see below, Fig. S11-S12).

We note that while these systems can offer sub-micrometre spatial resolution data (290 nm) (Fig. S4), they do make a trade-off between mass resolution and sensitivity by pulsing the acquisition rather than the source. This approach results in reduced collection efficiency. However, for the purposes of this study, both sensitivity and mass resolution were more than adequate for discerning the features of interest. The mass resolution for $^{11}B^+$ was 590, the mass resolution for $^{23}Na^+$ was 814, the mass resolution for $^{52}Cr^+$ was 918, and the mass resolution for $^{56}Fe^+$ was 984. These conditions were sufficient for isotope mass separation. However, determining the effective dosage from the analysis is challenging due to the continuous illumination of the Ga ion source and the short pulse duration of the acquisition, which creates a substantial gap between the acquired dosage window and the actual illumination dosage. The *in situ* heating TOF-SIMS experiment was immediately stopped when the temperature reached 850 °C. The data sets were exported by the TofSimsExplorer software and saved as the 'h5' hierarchical data format.

***In situ* heating XRD experiments.** Complementary *in situ* heating XRD experiments were conducted using a PANalytical Empyrean (Malvern Panalytical) diffractometer equipped with an Anton Paar HTK16N heating stage with a 0.5 mm thick platinum (Pt) heating strip. The Pt strip height alignment was performed while monitoring an alignment tunnel in the direct X-ray beam. After the alignment, a bulk oxidized steel sample, measuring 3 mm in width and 10 mm in length and coated with sodium borate, was positioned and secured at the middle of the Pt strip. To minimize temperature differences between the bottom and top surfaces of the sample, the sample thickness

was prepared at 1 mm. The XRD measurement was performed using a Bragg–Brentano reflection geometry with Cu Kα radiation (λ = 1.5406 Å, 45 kV accelerating voltage and 40 mA current). All scans were performed in a 2θ angle range of 5–60° and a speed of 5°/min under vacuum conditions. The heating rate during temperature ramps was set to 25 °C/min with a minute wait at each target temperature in the measurement program to ensure consistent temperature distribution within the samples before exposing them to the X-ray beam. The data was exported, processed, and indexed by Highscore Plus software and the ICDD database.

Data processing for TOF-SIMS. The h5 TOF-SIMS data sets were exported and processed using Tofwerk software and Python-based scripting. The Tofwerk software was used for data screening and exporting mass spectra from the selected areas in the images and specific frames along the Z-direction. Alignment of mass spectra at each pixel position and within the Z-direction was achieved in Tofwerk using the $^{69}Ga^+$ peak for reference. Spectral shifts were approximated based on peak intensity and refined using a subpixel cross-correlation routine. When exported as the h5 file, the data file contains the peak list. The h5 file was loaded and unpacked using the h5py (version 3.8.0) Python package[7]. The peak list, containing information on peaks in the mass spectrum at each pixel position and individual temperature, was accessed and processed using the HyperSpy (1.6.5) Python packge[8]. Once the peak list was loaded, it was used for multivariate analysis using non-negative matrix factorization (NMF). NMF is one of several multivariate statistical approaches which aims to linearly decompose a data set into a simplified representation subject to particular assumptions or constraints. NMF operates under the physical assumptions that the data are non-negative and that the entire spectrum image can be separated into a few spectral factors and their corresponding maps. Prior to NMF analysis, data pre-treatment procedures were performed, following the workflow reported in Ref. [9]. This included normalizing map intensities by the total ion counts per pixel and removing intense Ga peaks to enhance the image contrast of the other elements and compounds. NMF analysis was carried out as reported previously using Hyperspy[10, 11]. NMF in Hyperspy uses a project gradient method[12], one of several possible implementation alternatives such as multiplicative update rules or alternating least-squares[13]. To determine the appropriate number of components to retain in NMF, a principal component analysis (PCA) decomposition was initially performed to generate a scree plot (Fig. S7). The scree plot was then used to assess the number of components or factors needed to explain the majority of the variance in the data. Subsequently, NMF was carried out using the number of components derived from PCA as a starting point. The number of components retained in NMF was then adjusted one at a time to ensure a physically meaningful factorization with minimal noise-associated factors. NMF factors are not orthogonal like principal components, providing an alternative, but more physically meaningful representation as PCA often returns components with negative signal contributions.

Following NMF analysis, NMF maps corresponding to the weights across the sample image for the spectral factors primarily displaying $^{23}Na^+$ peak were employed to measure the transformed segment

length *L* (μm), representing the diffusion distance of sodium in the oxide scale during isochronal heating. The measurement was performed using Fiji-ImageJ software. The diffusion distance of sodium was assessed from the top of the continuous oxide scale to the location where sodium diffused to in the oxide scale for individual temperatures (Fig. S2), ranging from 260 °C to 400 °C. This temperature range captures the range within which sodium diffusion initiated from the top to the bottom of the continuous oxide scale and corresponds to approximately 30-40 nm removed by sputtering by 30 kV Ga ions. As such, the micron-scale features do not change significantly, but the surface is continuously removed to reveal previously buried material (*L* reports on interior propagation of the front). Through positioning the boxes, we have sought to ensure that the top of each box is aligned as precisely as possible with the interface between the two oxide layers. The starting point (x_0) for the measurement is taken at the boundary between the dendrite oxide layer and the continuous oxide layer, marked in Fig. S2 by a white dash line. The sodium diffusion distance is measured from the position $x_{i,}$ where the intensity of sodium falls abruptly (Fig. S2), to the position x_0. This approach may introduce some uncertainty where the interface between two oxide layers is not entirely flat. The main effect in a region with a convex interface would be to slightly underestimate the average value of *L* as the propagating front would spread out laterally in addition to the linear direction selected. Conversely, for a concave interface the effect would likely lead to an overestimated *L*. Where the interface curvature is small in the vicinity of the selected boxes (and by combining multiple boxes with local variations in interface non-linearity) we expect these effects to provide a reasonably unbiased estimate of *L*. The estimated error (1 standard deviation) for *L* is <210 nm or <3 pixels (78 nm pixels) across the temperature range used for the diffusion analysis. Based on established characteristics of liquid metal ion source Ga FIBs operated at 30 kV,[14] a 230 pA ion beam current produces a minimum obtainable probe size characterized by a d_{50} value (diameter containing 50% of the ion beam current) of 20-30 nm. Half the ion beam current extends beyond this characteristic diameter, likewise producing SIMS signals in a larger area. At a given signal-to-noise ratio, the obtainable spatial resolution is a further separate figure of merit, which we estimate at 290 nm (Fig. S4), though this estimate is conservative as it is defined from an interface that may not be perfectly sharp. Taken together, the defining spatial resolution is the largest length scale for separating features. However, the precision of feature localization will be much higher as indicated by the smaller spread in values of *L*. Beam size in this case does not appear to make a predominant contribution to uncertainties in *L*.

Prior to the measurement, image alignment accounting for the thermal drift occurred during *in situ* TOF-SIMS heating was performed to remove spurious effects from image drift. Filtered cross-correlation approach was used to estimate the shift matrix[15] followed by applying this matrix to the dataset using the function align2D() in the Hyperspy. Four different areas within the image were selected for the measurement (Fig. 4), and the results were averaged to estimate experimental uncertainty in the measurement. The temperature-dependent sodium diffusion length was further analysed by nonlinear least-squares curve fitting, following equation (1) or (S8). Curve fitting was

implemented using the curve_fit function in the SciPy Python package, with additional evaluation of fitting quality using the determination coefficient R^2, the reduced chi-square statistic χ^2, and by inspection of a histogram of the residuals (see below).

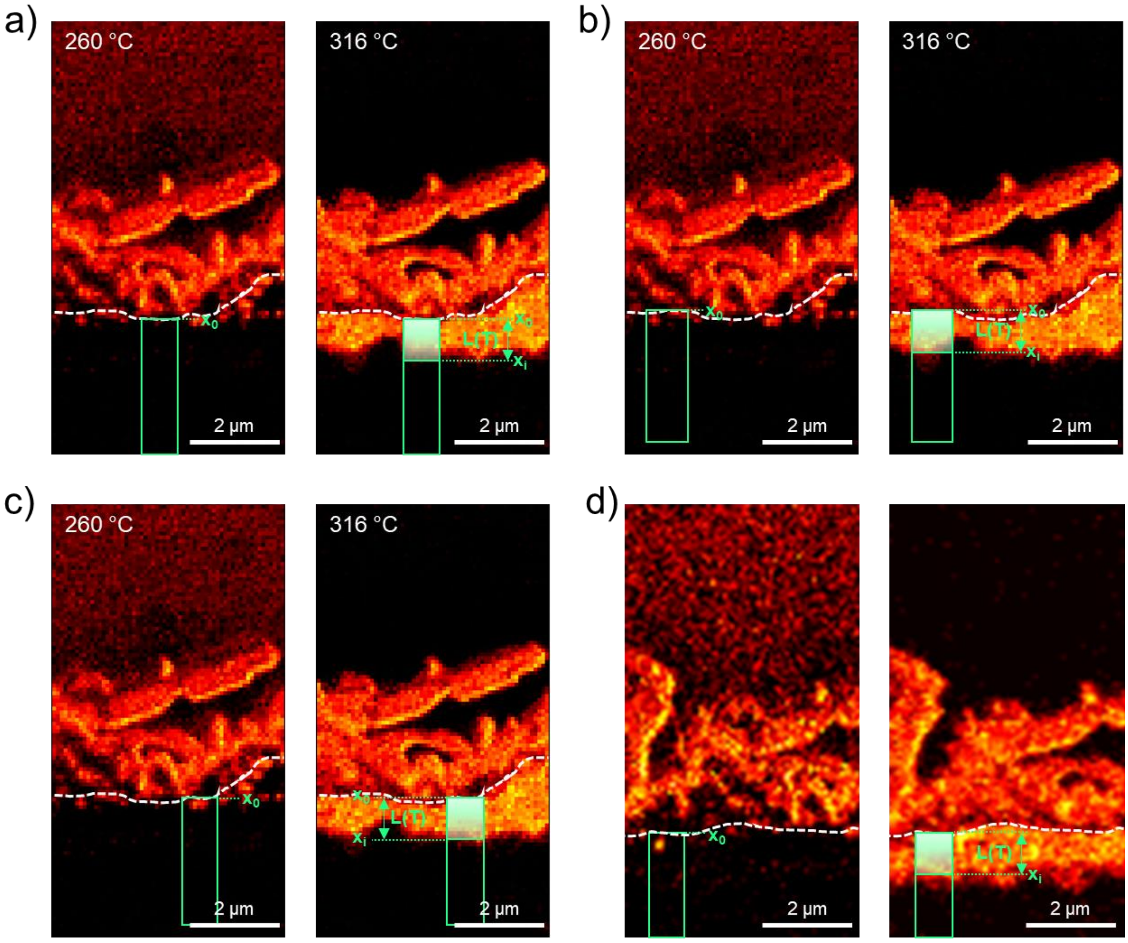

Figure S2. (a)-(d) The measurement of a sodium diffusion distance in the continuous oxide scale for individual temperatures across four different areas.

In situ heating SEM experiments

Figure S3a-S3h, displaying backscattered electron images, shows the corrosion reactions between sodium borate coating and oxide scale during *in situ* heating. The coating softened at 520 °C, and started to dissolve and penetrate the oxide layer (Fig. S3c), earlier than its reported glass transition at 555 °C,[16] attributed to the vacuum conditions. At 590 °C, the oxide scale was dissolved significantly, and the coating exhibited liquid-like behaviour (Fig. S3d). The images show enhanced contrast in the coating above 590 °C, indicating the significant incorporation of Fe and/or Cr within the coating. Above 600 °C, fine particles precipitated in the coating (Fig. S3e-S3h) which were composite oxides of Fe and/or Cr with boron oxide, as previously reported[1, 17]. Simultaneously, corrosion attack of the steel occurred at 620-650 °C, as evidenced by the formation and propagation of cracks in the steel substrate (Fig. S3g-S3h). This real-time observation highlights intricate behaviour of the glass melt, requiring comprehensive understanding of the chemical dynamics governing the dissolution of oxide layer and precipitation of composite oxide particles prior to the hot corrosion attack.

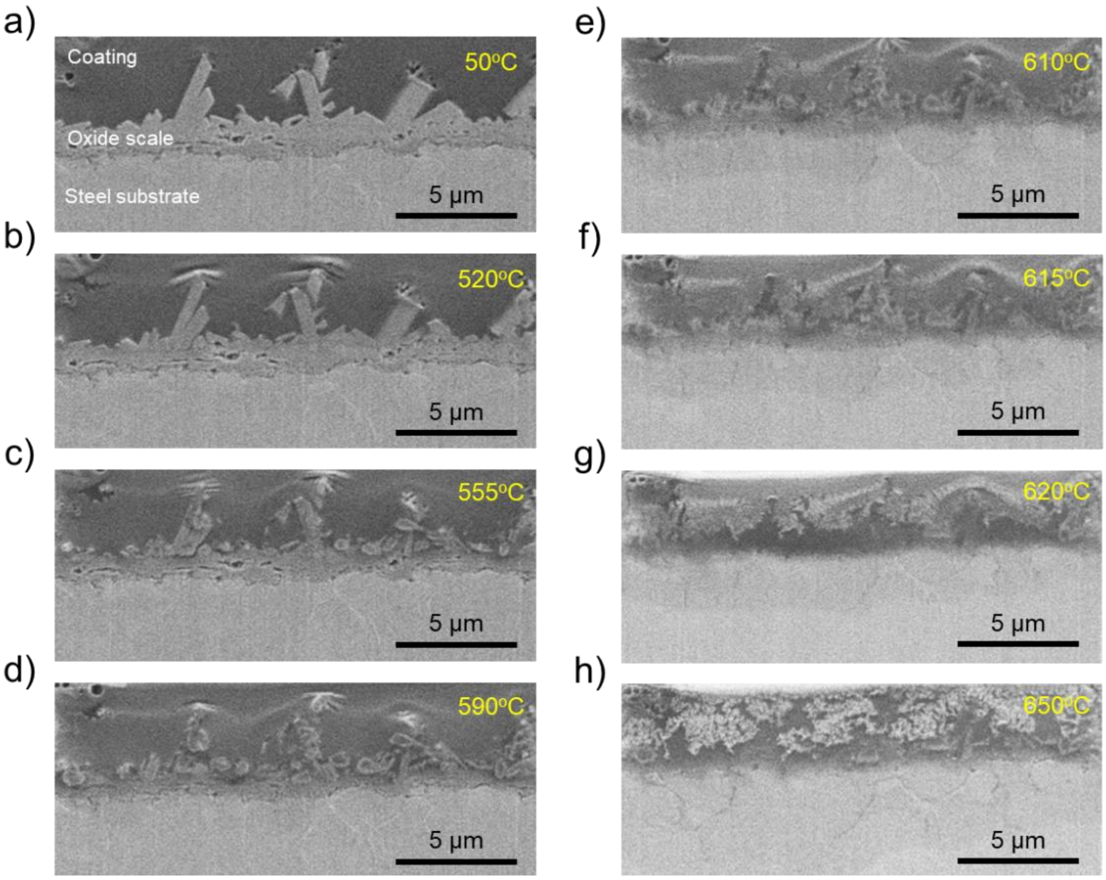

Figure S3. Cross-sectional backscattered electron temperature-series showing the hot corrosion reactions between sodium borate and the oxide layer in real time. (a)-(d) The softening of the borate coating and the dissolution of the oxide layer by the borate melts from 50-590 °C. (e)-(h) The precipitation of the composite oxides and the corrosion of the steel substrate by the borate melt above 600 °C. The changes in contrast of the coating layer in association with the increase of temperature, highlighting the incorporation of heavier metallic elements within the coating, is clearly observable.

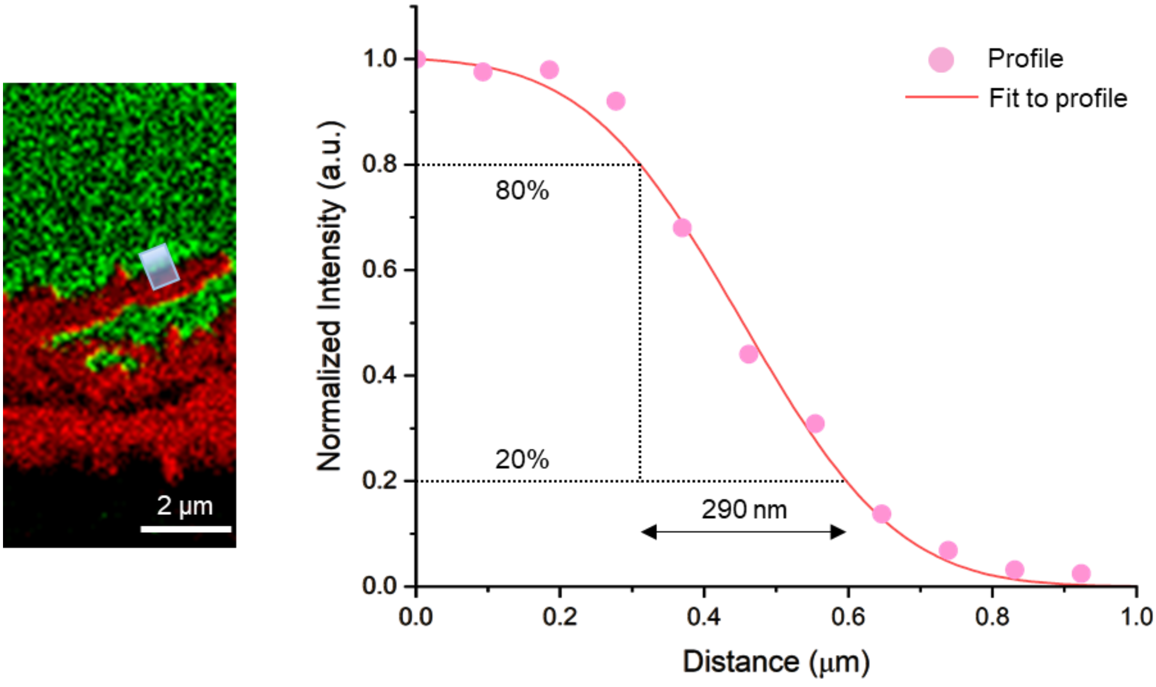

Figure S4. Intensity profile of $^{23}Na^{+}$ across the interface between the borate coating and the oxide particle, as marked in the image, used to estimate the lateral spatial resolution of an image, assuming an edge-on interface. The lateral resolution of an image, in general, depends upon the signal to noise ratio of the image, and is often defined as a distance along a line scan profile across a well-defined step function where the relative intensity changes between 20% and 80%[18]. By fitting the intensity profile of $^{23}Na^{+}$ across the interface to the complementary error function (the convolution of a Gaussian with a step function), the spatial resolution is estimated at 290 nm. A reasonable fit is obtained suggesting that the beam profile is well approximated by a Gaussian distribution which is reported as a good approximation to gallium ion beam spot distributions[19].

Evaluating ion beam condition for TOF-SIMS analysis

We investigated ion beam conditions and temperature effects on the signal-to-noise ratio, the mass resolution, and the intensity of specific secondary ions related to gallium ions. Our goal was to evaluate the sputtering parameters for *in situ* TOF-SIMS heating. Initially, we assessed signal-to-noise ratios using ion maps in Figure S5. The dataset collected with a low-energy ion beam (5 kV voltage, 68 pA current) was taken in a single exposure for fair comparison with *in situ* TOF-SIMS data extracted at the relevant temperature frame in the *in situ* heating TOF-SIMS dataset. The ion maps of $^{11}B^+$, $^{23}Na^+$, $^{52}Cr^+$, and $^{56}Fe^+$ collected under different ion beam conditions at room temperature (Fig. S5a-S5b) reveals the absence of $^{11}B^+$ and $^{23}Na^+$ in the coating layer. This is likely due to the resistance of glass materials to sputtering. However, the ion maps of $^{52}Cr^+$ and $^{56}Fe^+$ show enhanced signals, enabling comparison between beam conditions. Lower-energy beams produced significantly reduced signal intensities in the ion maps, aligning with the significantly lower intensity in peaks in the total mass spectrum (Fig. S6a). The total mass spectrum acquired at an ion beam voltage of 30 kV and ion beam current of 230 pA shows additional peaks albeit at low intensity, making this condition more suitable for detecting trace elements or compounds during solid-state reactions. However, the mass resolution of peaks acquired at this condition was slightly lower (538 for $^{11}B^+$, 694 for $^{23}Na^+$, 908 for $^{52}Cr^+$, and 902 for $^{56}Fe^+$) than those obtained at the 5 kV settings (674 for $^{11}B^+$, 784 for $^{23}Na^+$, 933 for $^{52}Cr^+$, and 984 for $^{56}Fe^+$).

The ion maps acquired at 200 °C showed detectable $^{11}B^+$ and $^{23}Na^+$ ions in the coating layer for both beam conditions (Fig. S5c-S5d), suggesting more favourable sputtering of glasses at high temperatures. Similarly, lower energy beam conditions produced weaker signal intensities in the ion map images, corresponding with lower intensities of peaks in the total mass spectrum (Fig. S6b). Intriguingly, mass resolutions for the higher energy beam conditions (590 for $^{11}B^+$, 814 for $^{23}Na^+$ for, 918 for $^{52}Cr^+$, and 984 for $^{56}Fe^+$) were better than those obtained at the lower ion beam energy settings under heating at 200 °C (544 for $^{11}B^+$, 740 for $^{23}Na^+$, 870 for $^{52}Cr^+$, and 812 for $^{56}Fe^+$). Besides, the ratios of $^{23}Na^+$ and $^{11}B^+$ peak intensities over $^{69}Ga^+$ ions were higher for the higher energy beam conditions, suggesting higher yield of these ions at this beam condition at high temperatures. Based on this evaluation, we determined that the ion beam condition of 30 kV and 230 pA was suitable for TOF-SIMS analysis during *in situ* heating. This condition provides a higher signal-to-noise ratio for mapping chemical transport as well as a higher signal intensity in the mass spectrum to detect the formation of any chemical phases at low overall sample fraction during solid-state chemical reactions. However, it is important to note that these conditions might introduce more Ga implantation and increase the sputtering rate of the sample materials.

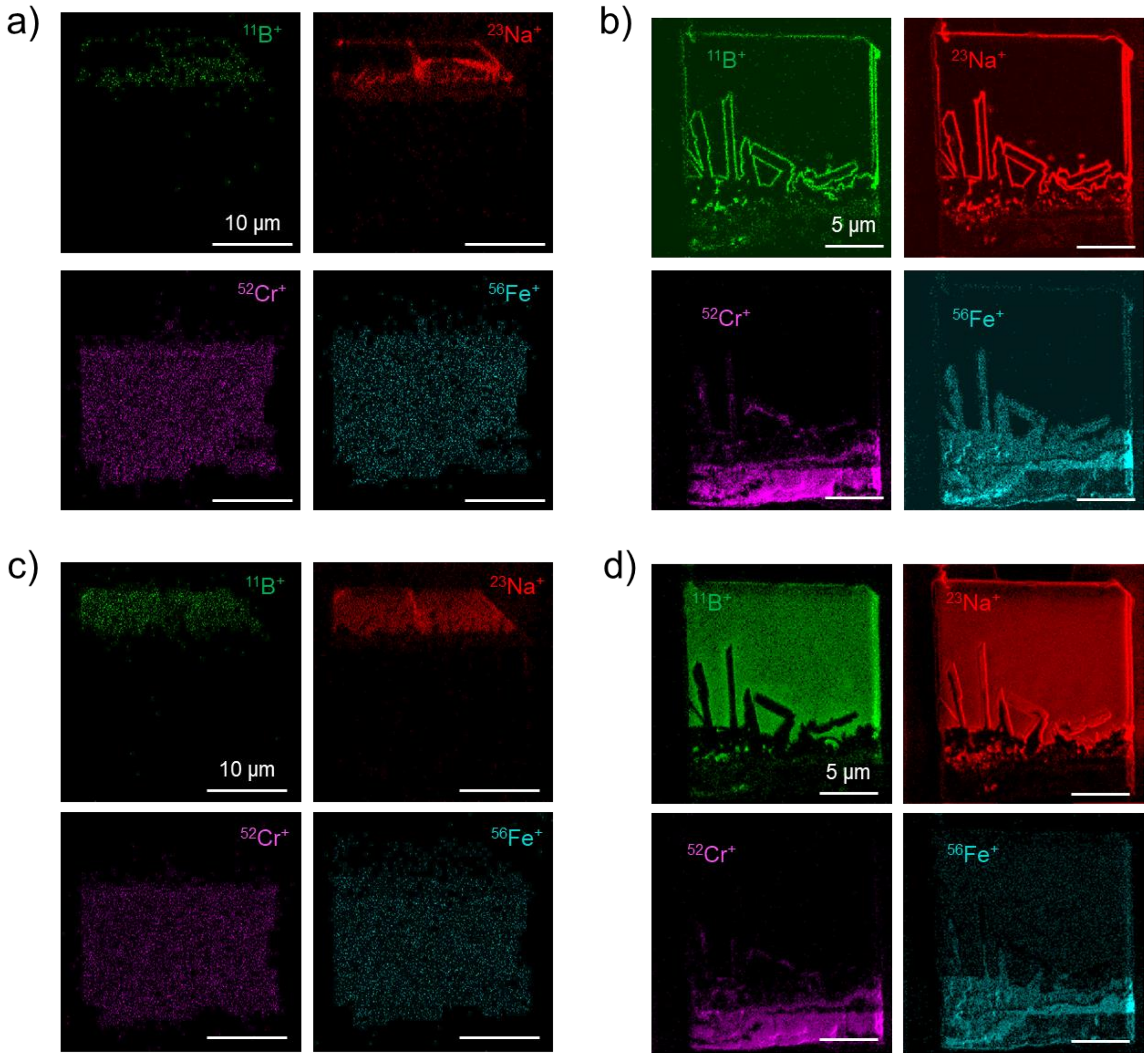

Figure S5. Effect of ion beam condition and temperature on the signal-to-noise ratio of the ion maps. Ion maps of $^{11}B^+$, $^{23}Na^+$, $^{52}Cr^+$, and $^{56}Fe^+$ were exported from the dataset acquired at: (a) room temperature, 5 kV voltage, and 68 pA current; (b) room temperature, 30 kV voltage, and 230 pA current; (c) 200 °C, 5 kV voltage, and 68 pA current; (d) 200 °C, 30 kV voltage, and 230 pA current.

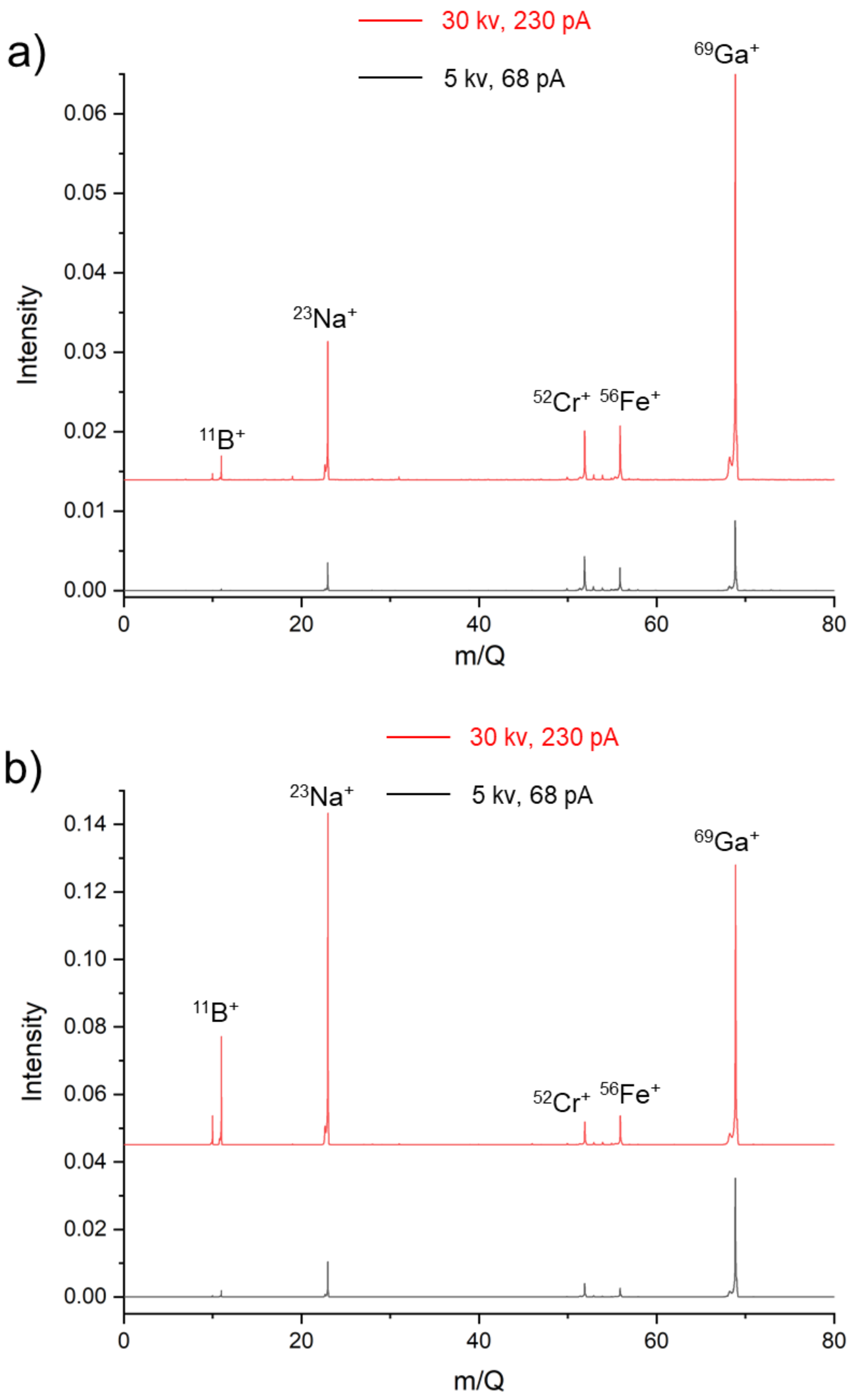

Figure S6. Effect of ion beam condition and temperature on the signal intensity of mass spectrum. (a) Comparison between total mass spectra acquired at room temperature but different ion beam conditions. (b) Comparison between total mass spectra acquired at 200 °C but different ion beam conditions.

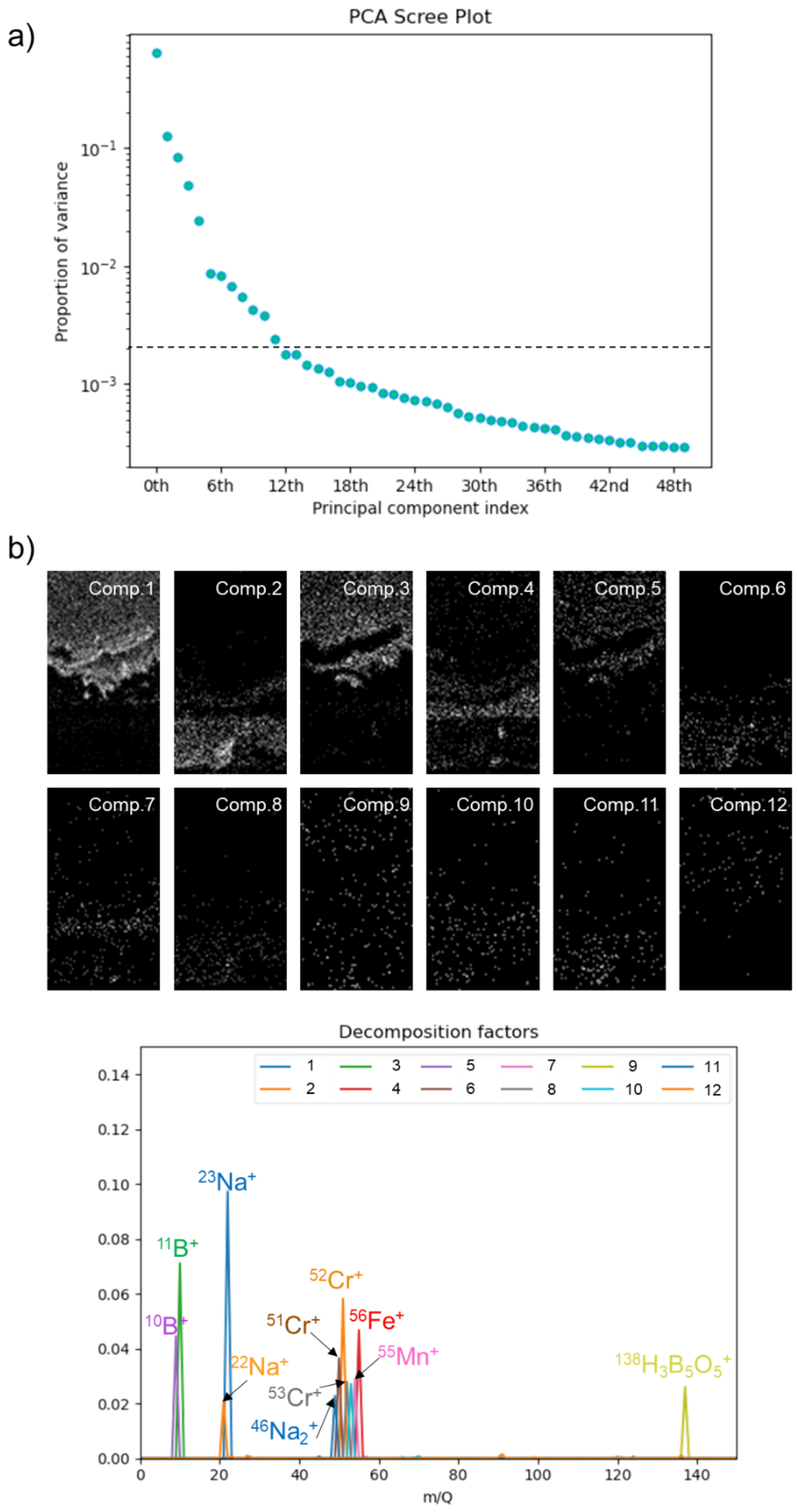

Figure S7. Example principal component analysis scree plot showing the proportion of variance associated with each of the first 50 principal components for the TOF-SIMS dataset shown in Figure 1b (a). The 'elbow' in the scree plot, as marked by the dashed line, was used to estimate the number of factors to retain in NMF analysis. In this instance, twelve principal components were indicated by the scree plot, and so factorization using NMF was initially explored in the neighbourhood of twelve factors (b). We found 8 factors that can be used to produce a physically meaningful factorization without including noise factors and other weakly contributing factors.

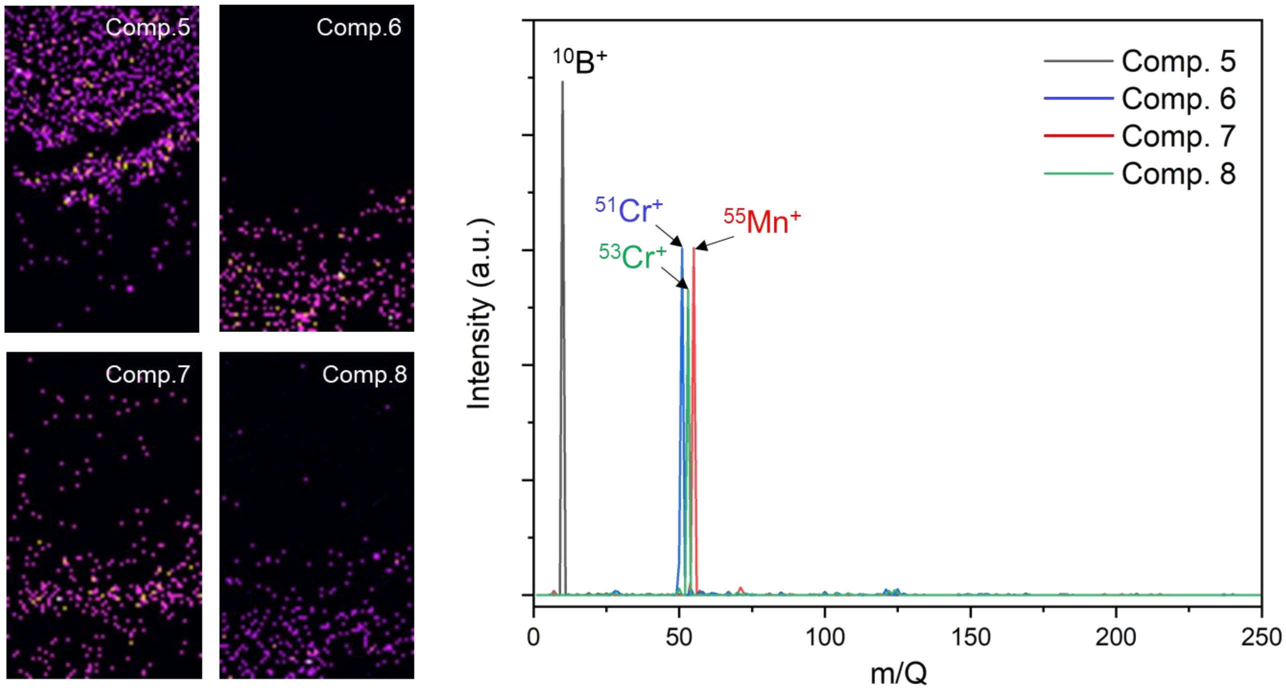

Figure S8. Other loading maps and corresponding spectral factors determined from NMF of *in situ* TOF-SIMS data at 250 °C. The maps shown in the figure were the additional four components associated to the spectral factors that show primarily $^{10}B^+$, $^{51}Cr^+$, $^{53}Cr^+$, and $^{55}Mn^+$.

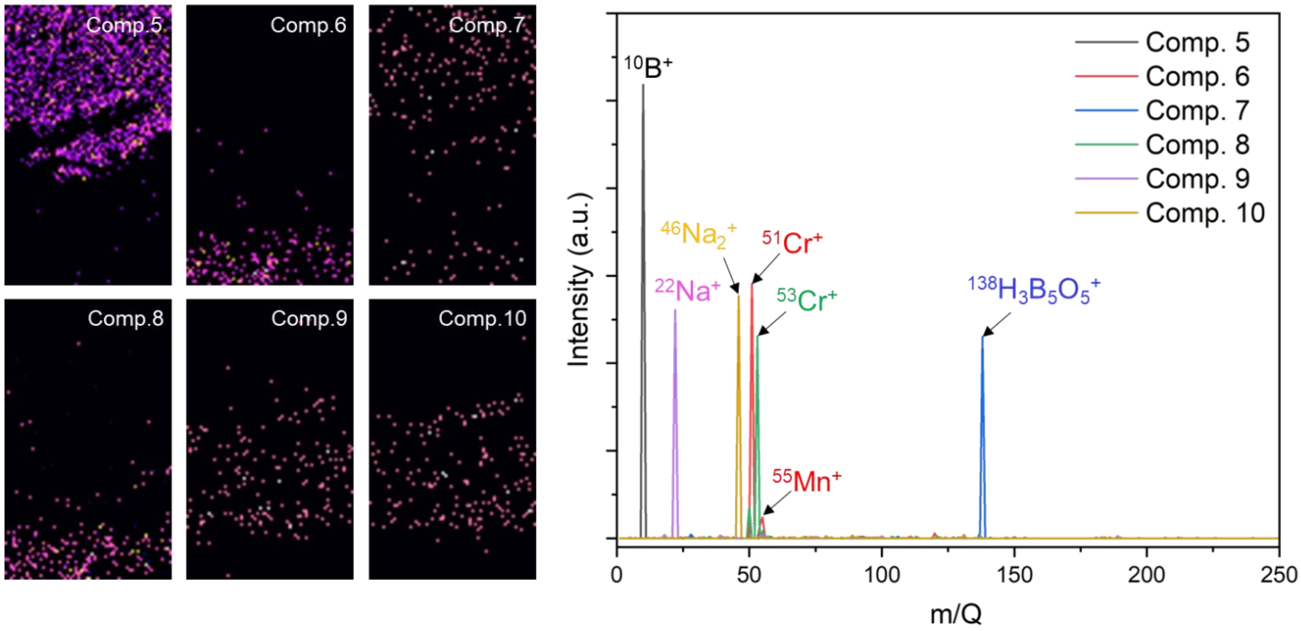

Figure S9. Other loading maps and corresponding spectral factors determined from NMF of *in situ* TOF-SIMS data at 350 °C. The maps shown in the figure were the additional six components associated to the spectral factors that show primarily $^{10}B^+$, $^{22}Na^+$, $^{46}Na_2^+$, $^{51}Cr^+$, $^{53}Cr^+$, $^{55}Mn^+$, and $^{138}H_3B_5O_5^+$.

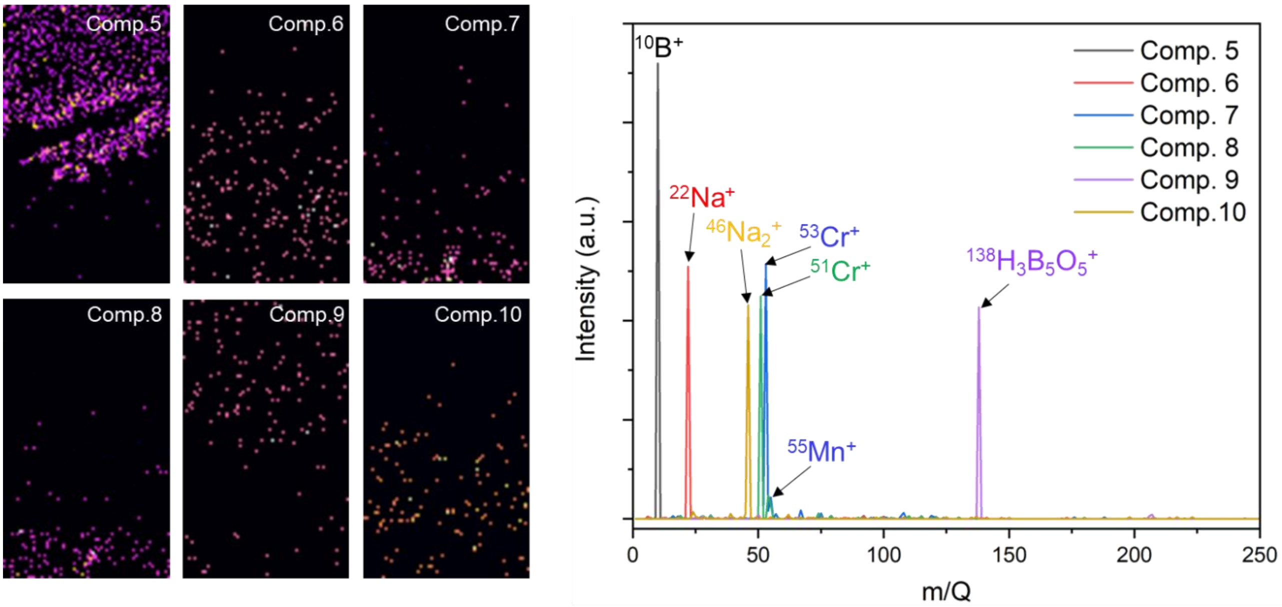

Figure S10. Other loading maps and corresponding spectral factors determined from NMF of *in situ* TOF-SIMS data at 420 °C. The maps shown in the figure were the additional six components associated to the spectral factors that show primarily $^{10}B^+$, $^{22}Na^+$, $^{46}Na_2^+$, $^{51}Cr^+$, $^{53}Cr^+$, $^{55}Mn^+$, and $^{138}H_3B_5O_5^+$.

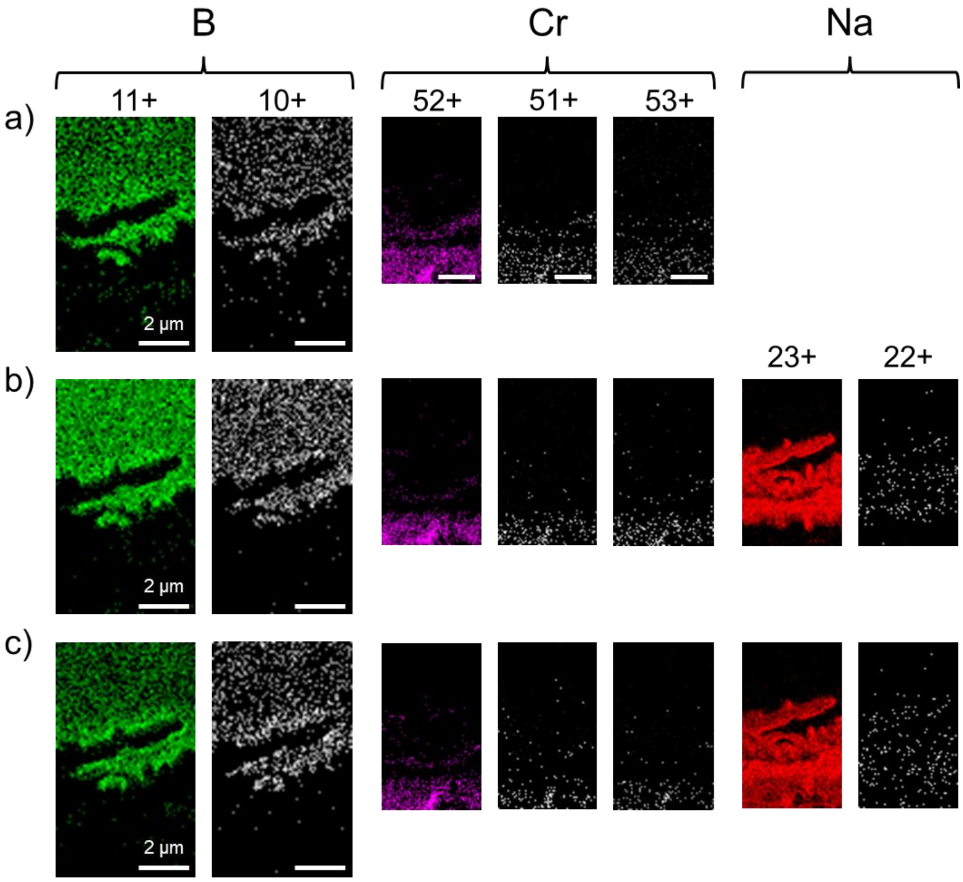

Figure S11. Spatial distribution of the isotopes B^+, Cr^+, and Na^+ isotopes as mapped by NMF analysis for (a) 250 °C, (b) 350 °C, and (c) 420 °C.

Evaluating the effects of ion beam irradiation on chemical transport

TOF-SIMS is one of a small number of techniques that can provide depth and lateral chemical information about materials. There is an increasing number of studies using TOF-SIMS as a primary technique to investigate changes in chemical structures *in situ*[20, 21]. Recent advances in coupling TOF-SIMS with FIB-SEM allow for chemical analysis at higher spatial resolution, opening a pathway to study materials with TOF-SIMS at small length scales[22]. While there is no doubt that the use of FIB-SEM-SIMS will become more popular in materials research, there are concerns about acquiring reliable data that need to be carefully considered. In the study of Harvey et al.[23], a TOF-SIMS measurement artefact was reported, noting the apparent A-site cation gradient typically seen in TOF-SIMS depth profiles and often ascribed as an intrinsic feature of perovskite photovoltaics. This artefact is due to beam damage from primary ion beam that changes the chemical structures of perovskite materials. Other studies[24, 25] have also revealed the effect of ion beam irradiation on the chemical changes in layered ferroelectric semiconductors and organic photovoltaic heterojunction layers by TOF-SIMS analysis.

In this study, repeated scanning with gallium (Ga) ion beams during *in situ* heating experiments may induce some effects on chemical transport. The replacement collision sequences associated with the sputtering process could create an excess of vacancy defects in the sub-surface region, serving as active sites to trap charge carriers[26]. Meanwhile, potential implantation of Ga can also induce self-atom mixing in crystalline and amorphous materials[27]. These effects might potentially influence the kinetics of the chemical processes observed, and hence, changing the nature of the chemical transports. Moreover, since SIMS is surface/sub-surface sensitive technique, another question arises whether the sodium diffusion seen in this study are representative of what occurs in the bulk samples. Our previous studies[1, 2, 17, 28] have seen the diffusion of sodium from inorganic glasses to the oxide layers at elevated temperatures via post-mortem cross-section analysis of the samples suggesting the diffusion of sodium is an intrinsic nature of the studied material systems. However, the diffusion of sodium below the glass transitions of the inorganic glass coatings in this study warrants further validation as it may be influenced by beam-induced artefacts.

To validate the results, we have sought to compare the *in situ* TOF-SIMS data observed with the data obtained from controlled TOF-SIMS analysis at individual temperatures (Fig. S12). For control experiments, the specimen was heated to a certain temperature and the TOF-SIMS data acquisition was carried out under a single exposure. This approach served to limit the ion beam irradiation on the specimen, limiting the effects of both defect vacancies formation and Ga implantations. The results show strong agreement in the observation of sodium diffusion, as seen in the different spatial distribution of sodium between the data acquired at 200 °C and the data acquired above 300 °C (Fig. S12b). In a further control experiment, the specimen was heated directly to 400 °C from 200 °C, held at 400 °C for 10 seconds, and cooled prior to the TOF-SIMS analysis. This control experiment was carried out at an ion beam voltage of 5 kV and an ion beam current of 68 pA (Fig. S13). Multiple

frames were collected and integrated for TOF-SIMS analysis conducted at this condition to improve the signal to noise ratio. Taken together and in comparison with the results in Figure 1 and the results in Figure S5a and S5c show the diffusion of sodium from the coating layer to the oxide and steel areas as well as incorporation of iron in the borate coating layer unambiguously.

Additionally, we also performed *in situ* XRD heating experiments in matched environmental conditions (Fig. S14) to corroborate the formation of phases occurring below the melting point of sodium borate. The sodium borate coating, comprising the original structure of $Na_2B_4O_7.10H_2O$, exhibited strong diffraction peaks. As the temperature surpassed 200 °C, a significant reduction in the intensity of these peaks was observed. We interpret this decrease in terms of loss of crystallinity which can be attributed to water evaporation and the structural decomposition of $Na_2B_4O_7$ into $Na_2O.B_2O_3$ glass[1, 28]. There are several peaks corresponding to iron oxides, although these were relatively weak, and there are no detectable peaks of chromium oxides. The low intensities of metal oxide peaks arise from their low concentration in the bulk samples compared to the contributions from the steel content and the sodium borate coating. Notably, the XRD patterns collected above 400 °C exhibited increased peak intensities at 33.9°, 35.1°, and 35.9°, which can be attributed to $NaFeO_2$ (ICDD 01-082-2834). This observation strongly supports the occurrence of the partial dissolution of hematite (Fe_2O_3) below the melting temperature, aligning well with the findings from the *in situ* TOF-SIMS analyses (Fig. 3c-3d). Furthermore, a close examination of chemical transport in Video 1 reveals the partial liquid flow of the top coating, evidenced by the initial decrease in boron intensity between 400-500 °C followed by a gradual increase in boron signals above 520 °C. This behaviour likely indicates material flow as the coating softened, leading to an intermixing of coating materials at the front and rear of the specimen. Importantly, while this intermixing occurred, the top layer still exhibited dominant boron intensity and a noticeable absence or reduced sodium intensity. This suggests that the diffusion of sodium from the coating to the oxide layers took place not only at the sputtering surfaces but also within the bulk of the specimens. Our observations from *in situ* XRD and *in situ* TOF-SIMS further demonstrate a set of consistent chemical dynamics occurred inside the bulk material probed by XRD and on the analyzed surface probed in ToF-SIMS. While surface diffusion rates may differ to those in the bulk, these control experiments indicate the ToF-SIMS analyses report predominantly on the bulk processes and bulk mobility of sodium ions. In conclusion, we believe that the chemical transport observed in this study represented an intrinsic characteristic of the studied material with minimal influence from the ion beam irradiation during TOF-SIMS analysis.

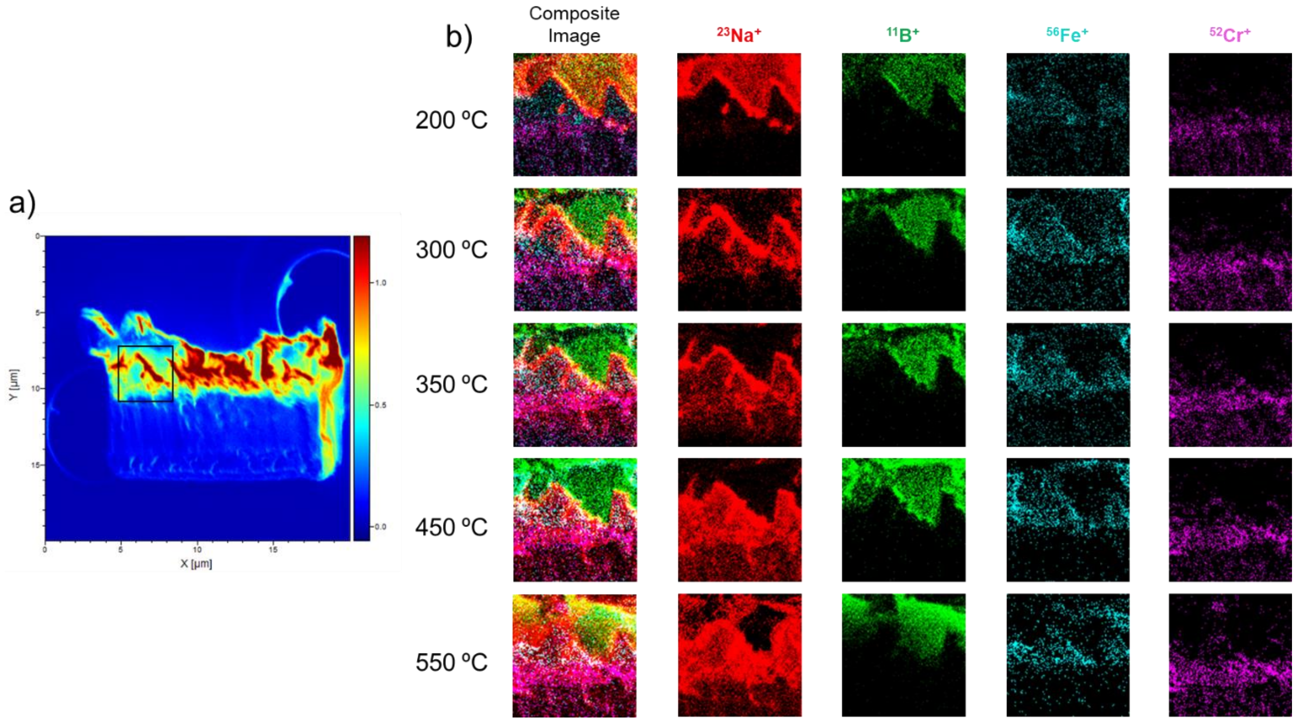

Figure S12. Single exposure TOF-SIMS analysis at individual temperatures showing a diffusion of sodium above 300 °C. The experiments were conducted by heating the specimen directly to the targeted temperatures (1 °C/second heating rate) followed by performing a single exposure TOF-SIMS analysis at 30 kV voltage and 230 pA current. (a) Overview image of the specimen being studied. The inset square box marked the area for visualization. (b) Ion maps of interested chemical elements, exported from TOF-SIMS data acquired at 200 °C, 300 °C, 350 °C, 450 °C, and 550 °C.

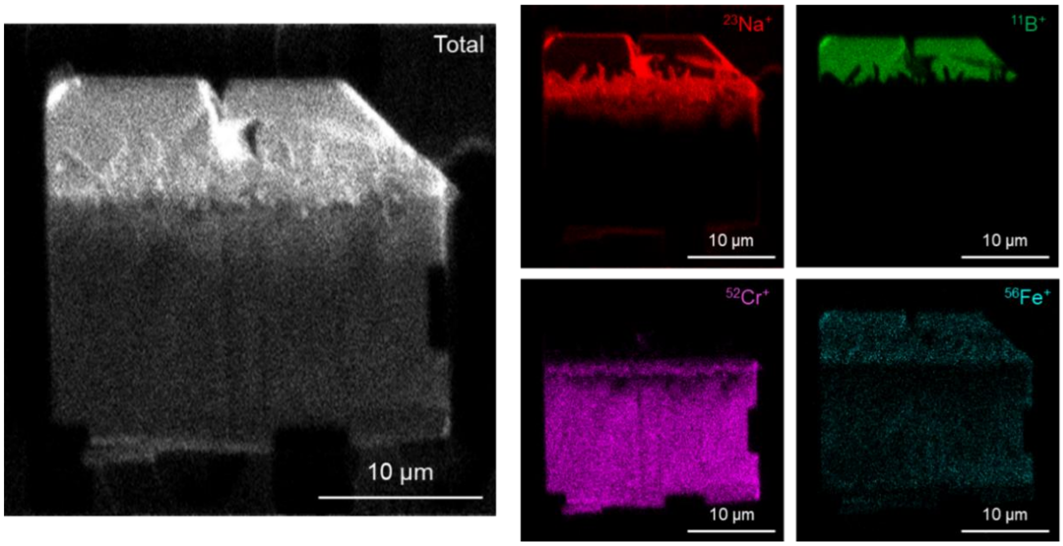

Figure S13. Multi-frame TOF-SIMS analysis of the post-mortem specimen after heating the specimen to 400 °C from 200 °C followed by cooling the specimen to room temperature. The TOF-SIMS analysis was conducted at 5 kV voltage and 68 pA current. The images were constructed by integrating multiple frames to enhance signal-to-noise ratio at this ion beam conditions.

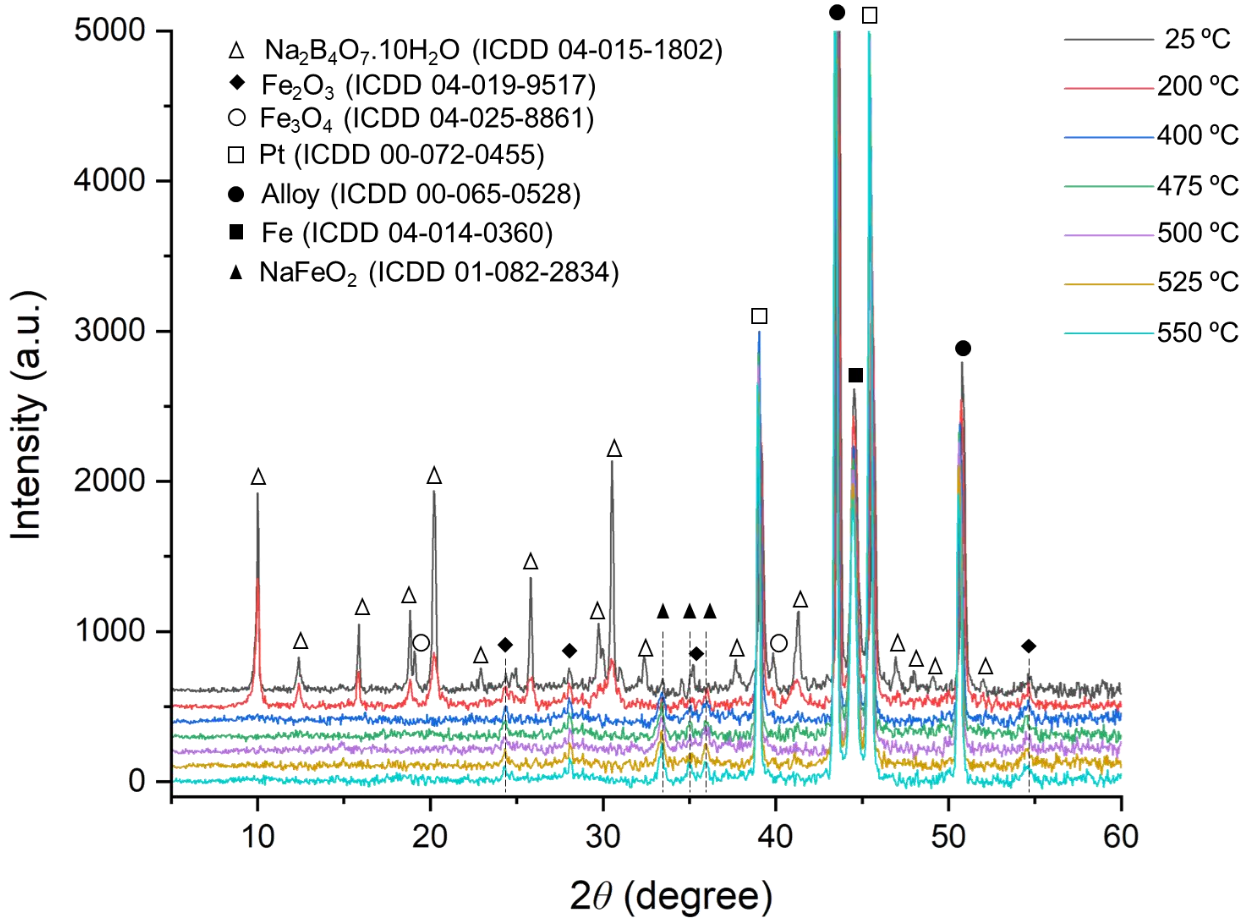

Figure S14. *In situ* XRD heating results showing dynamic phase formation during solid-state chemical reactions between sodium borate and oxides.

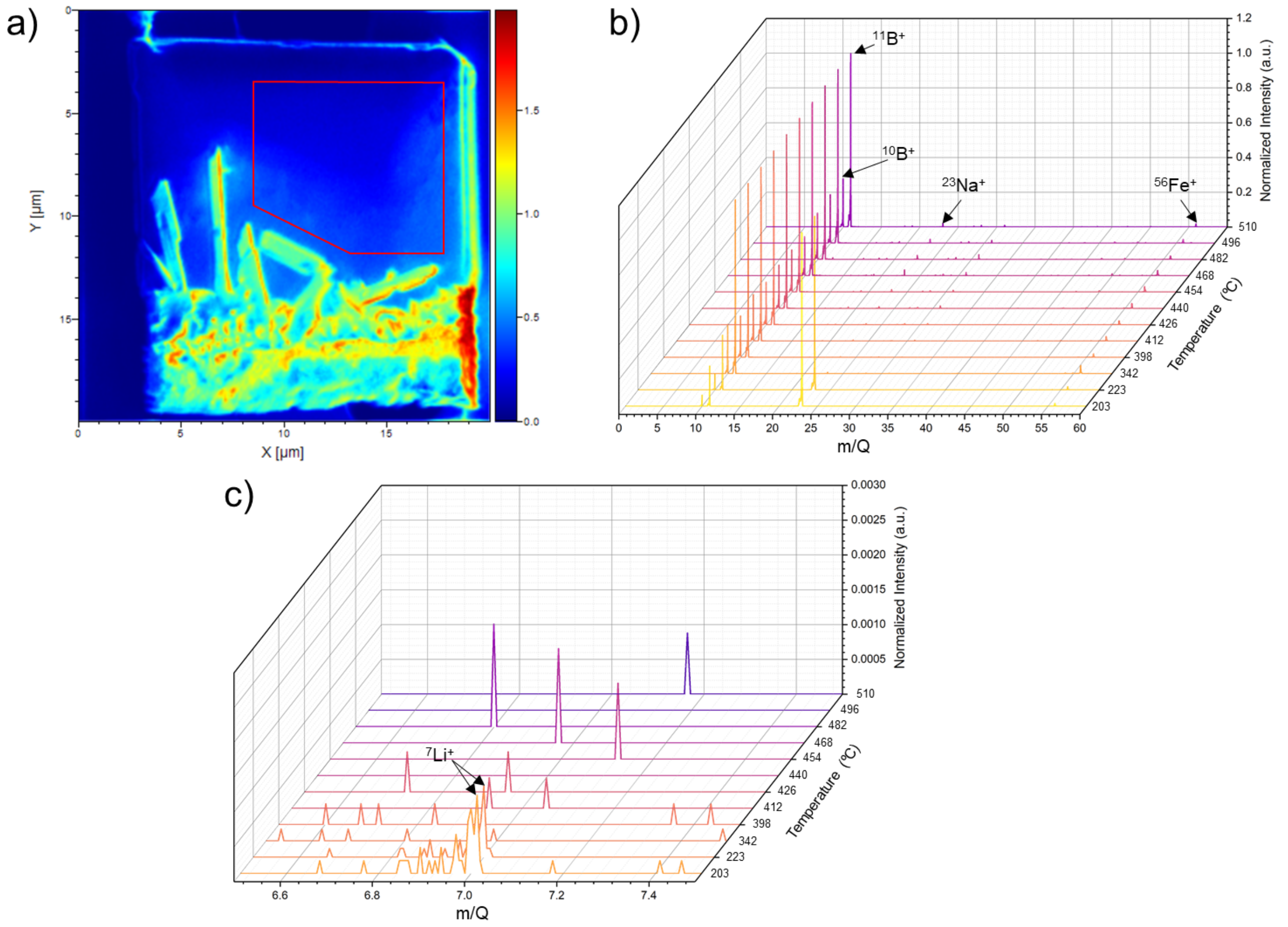

Figure S15. Dynamic chemical changes in the borate coating as examined by the mass spectra at individual temperatures. (a) Overview total ion image showing the red marked area where the mass spectra was extracted. (b) Mass spectra at individual temperatures used for analysis of sodium diffusion. (c) Cropped representations of the spectra shown in (b) depicting a reduced m/Q range to highlight the peak intensity of $^{7}Li^{+}$, indicating that trace amounts of lithium were present in the borate coating before the diffusion occurred.

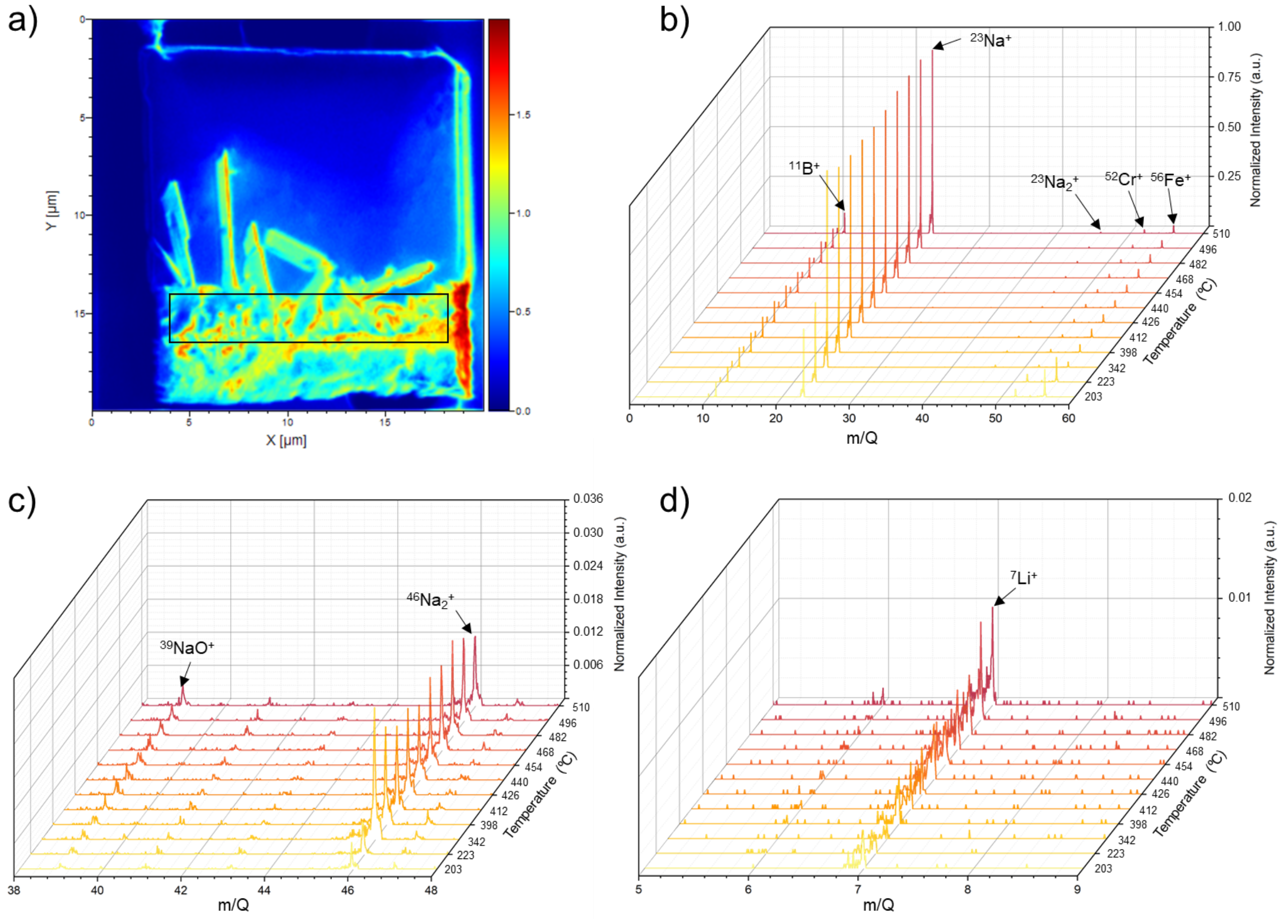

Figure S16. Dynamic chemical changes in the iron oxide layer as examined by the mass spectra at individual temperatures. (a) Overview total ion image showing the black marked area where the mass spectra was extracted. (b) – (d) The mass spectra at individual temperatures where sodium diffusion took place. The mass spectra shown in (c) and (d) were cropped at specific m/Q ranges in (b) to reveal changes in peak intensity of some secondary ions contributing weakly to the total mass spectrum.

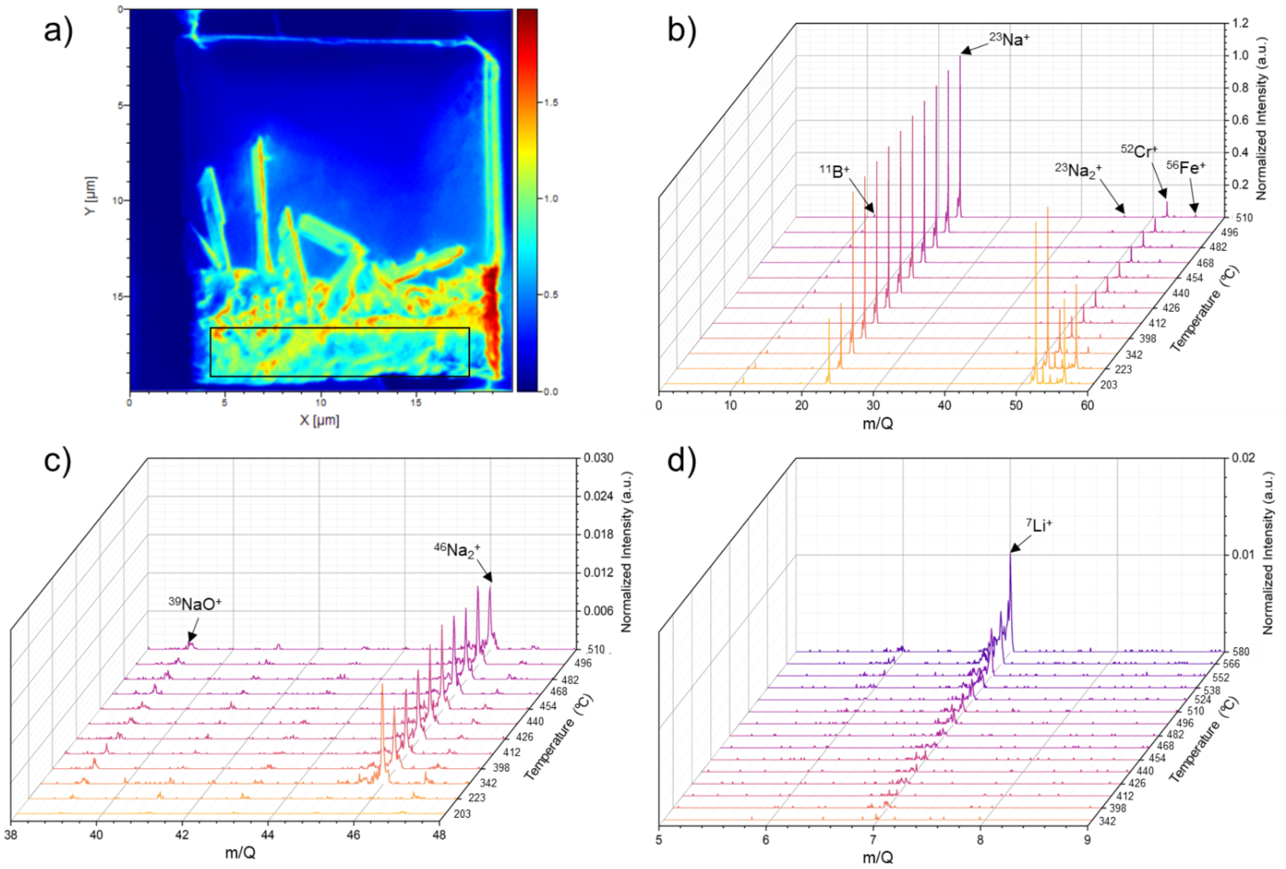

Figure S17. Dynamic chemical changes in the iron-chromium oxide layer as examined by the mass spectra at individual temperatures. (a) Overview total ion image showing the black marked area where the mass spectra was extracted. (b) – (d) The mass spectra at individual temperatures where sodium diffusion took place. The mass spectra shown in (c) and (d) were cropped at specific m/Q ranges in (b) to reveal changes in peak intensity of some secondary ions contributing weakly to the total mass spectrum.

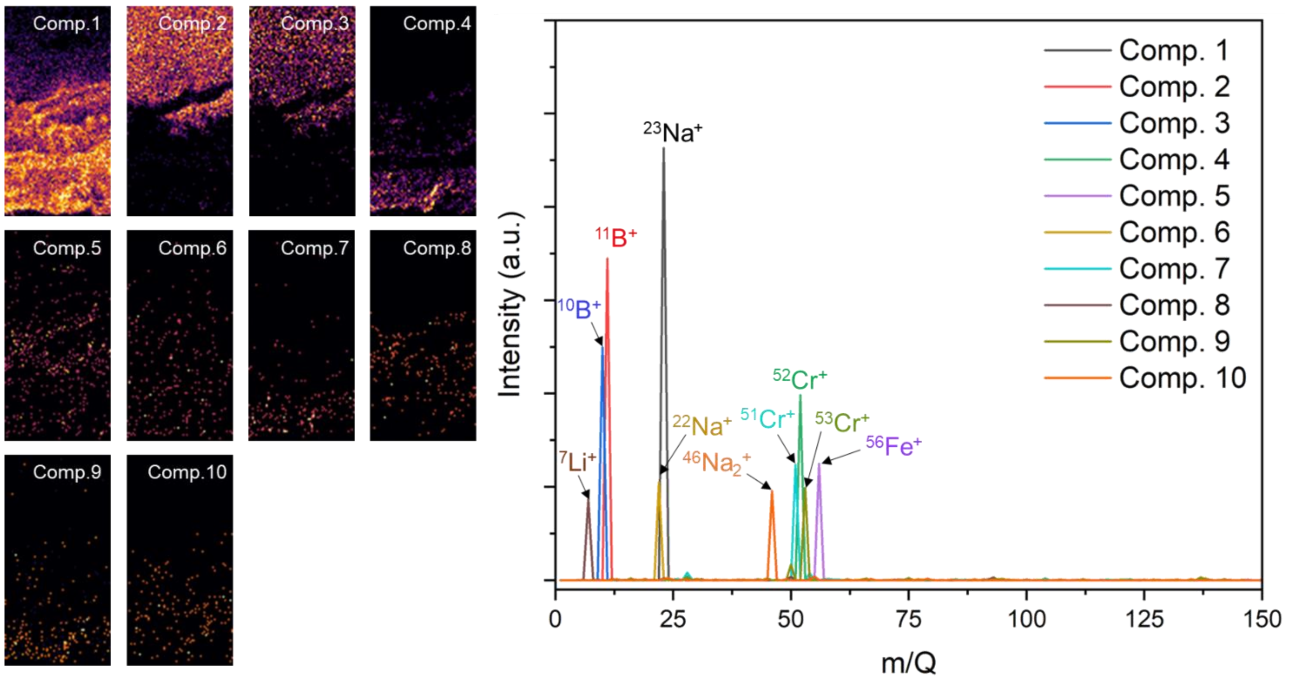

Figure S18. Loading maps and corresponding spectral factors determined from NMF of *in situ* TOF-SIMS data at 580 °C. 10 factors were identified that can be used to describe the dataset, although the signal intensity from component 5 to component 10 are weakly presented. Component 8 with its spectral factor primarily showing $^{7}Li^{+}$ displays the spatial distribution unambiguously within the oxide regions albeit at low signal-to-noise ratio.

Model for sodium diffusion length determination using *in situ* heating TOF-SIMS

To investigate the kinetics of sodium diffusion, we followed the progress of sodium diffusion in continuous oxide layers as a function of temperature. The diffusion of chemical elements within solid-state materials is often described by Fick's laws of diffusion[29]. We thus modelled the transformed segment length, L (μm), representing the diffusion length of sodium in solid oxides, as a diffusion-controlled change in the distribution of sodium. Two phenomena could occur during the diffusion of individual chemical elements in solid state materials[30]. The first pertains to diffusion across continuous areas where the chemical potential gradient remains constant, and the diffusion flux is proportional to the diffusion coefficient and the thickness (length or distance in cross-section) of the solid materials. The second phenomenon relates to interfacial diffusion between grains, with the flux being proportional to the interfacial rate constant and the difference in chemical potential across the interface. For simplicity, we measured the diffusion length of sodium within the continuous oxide layers and assumed that the analysed areas were homogeneous and contained no grain boundaries. In this instance, Fick's laws of diffusion can be used to derive a fitting function for the transport of sodium in the solid oxide layers.

Fick's law of diffusion describes the flux of a substance through a medium, resulting from concentration differences[29]. Fick's first law defines the diffusion of a substance in a stationary medium, assuming a constant diffusion coefficient and equating the flux entering the system with the flux exiting it. Meanwhile, Fick's second law provides a more comprehensive description of diffusion by incorporating the effects of time and spatial variations, revealing how the concentration of a substance changes over time and in space. In practice, Fick's second law is better suited to experimental systems where conditions are not steady. Particularly, Tyurnina et al.[31] and Schoo et al.[32] indicated that the diffusion of sodium in glasses and steel adheres to Fick's second law of diffusion by analysing the sodium penetration profile in solid materials. In this study, we employed Fick's second law to derive a fitting function for the sodium diffusion front. However, due to the isochronal heating process, the diffusion coefficient is not constant but dynamically changes with temperature (or time). Assuming the diffusion occurred in one dimension, we thus used the thin film solution of Fick's second law with a diffusion coefficient varying with time t and position x[33, 34]:

$$c\ (x,t) = \frac{M}{\sqrt{4\pi.D(t).t}} \exp\left(-\frac{x^2}{4.D(t).t}\right) \quad \text{(S1)}$$

Here, $c\ (x,t)$ is a local concentration at position x and time t, $D(t)$ is the diffusion coefficient, and M is constant and represents the total amount of the chemical element. If we consider the ratio of $c\ (x,t)/M$ to be a probability of observing a particle within a small one-dimensional volume element, with dx centred at x, we can write the continuous probability distribution for diffusion in one dimension as:

$$P\ (x,t)\ dx = \frac{1}{\sqrt{4\pi.D(t).t}} \exp\left(-\frac{x^2}{4.D(t).t}\right) dx \qquad \text{(S2)}$$

The root-mean-square displacement, $x_{rms} = \sqrt{\langle|x(t) - x_0|^2\rangle}$, shows the average distance to the centre of the moving path and therefore holds information about the transformed segment length, L (μm), for sodium diffusion. The time evolution of this length should then provide information about the rate of diffusion and can be related to the probability $P\ (x,t)$ over the position x as follow:

$$\langle|x(t) - x_0|^2\rangle = \int_{-\infty}^{+\infty} x^2.P\ (x,t)\ dx = \int_{-\infty}^{+\infty} \frac{x^2}{\sqrt{4\pi.D(t).t}} \exp\left(-\frac{x^2}{4.D(t).t}\right)\ dx \qquad \text{(S3)}$$

With the assumption that $D(t)$ does not change with location (position) x during the diffusion of the chemical elements, we can derive a simple solution for x_{rms} as below:

$$x_{rms} = L = \sqrt{2.D(t).t} \qquad \text{(S4)}$$

In our study, the heating rate is 1°C/second and the starting temperature for acquiring *in situ* TOF-SIMS results was 50°C. We can then relate time t as a function of temperature by a simple equation:

$$t\ (sec) = T(°\mathrm{C}) - 50 \qquad \text{(S5)}$$

Substituting equation S5 to S4, we have a function describing the change of transformed segment length, i.e. diffusion length of sodium, to temperature:

$$L = \sqrt{2.D(T).(T - 50)} \qquad \text{(S6)}$$

Since diffusion is a thermally-activated process, the diffusion coefficient, representing diffusivity, can be also described by an Arrhenius relationship[29]. The moving of the chemical elements in the solid-state materials requires an energy for diffusion to occur, normally known as an activation energy, and it can be related to the temperature by the following equation:

$$D(T) = D_0 . \exp\left(-\frac{Q_d}{R.(T+273)}\right) \qquad \text{(S7)}$$

where D_0 (μm^2/s) is a pre-exponential constant, representing the diffusion rate at infinite temperature, Q_d (J/mol) is an activation energy for the diffusion to occur, R is gas constant (~8.314 J.mol^{-1}.K^{-1}), and $T(°\mathrm{C})$ is temperature. Substituting equation S7 to S6, we have a function connecting the diffusion length of sodium to temperature in this study:

$$L = \sqrt{2.D_0.\exp\left(-\frac{Q_d}{R.(T+273)}\right).(T - 50)} \qquad \text{(S8)}$$

To evaluate the goodness-of-fit of the proposed model to the experimental data, we used goodness-of-fit statistics together with inspection of the residuals and evaluation of uncertainties in parameter estimation from the fit. For goodness-of-fit statistics, we used the coefficient of determination R^2 and the reduced chi-square (χ^2) test statistic. The R^2 can be defined from the sum of squared residuals

(SS_{res}) and the total sum of squares (SS_{tot}) which can be served as an indicator of the match between experiment and the fitted curve. Where the predicted values determined from the fitting model exactly match the measured values, the SS_{res} = 0 and R^2 = 1. Meanwhile, the χ^2 statistic can be used to evaluate a fitted curve in the context of variance in experimental measurements. For the purposes of the evaluation of curve fitting, the chi-square per degrees of freedom or reduced chi-square statistic is useful as it takes into consideration the number of data points (measurements) and number of parameters describing the fitted function. A model curve that fits the data within the variance inherent in the data will exhibit a χ^2_{red} near 1. A χ^2_{red} much greater than 1 will indicate a poor agreement between the model and the measurements, and a χ^2_{red} less than 1 will indicate the model fits more closely to the data than expected for the inherent variation in the measurements. A summary of the fitting coefficients are given together with the R^2, the number of data points N, the degrees of freedom v and χ^2_{red} in Table S1.

Table S1. Summary of the fitting coefficients and good-ness-of-fit statistics.

$\boldsymbol{D_0}$ (μm²/sec)	$\boldsymbol{Q}$ (J/mole)	$\boldsymbol{R^2}$	$\boldsymbol{N}$	**v**	$\boldsymbol{\chi^2}$
82327.1	88105.0	0.98	31	29	1.99

While R^2 value of 0.98 suggests a strong alignment between the proposed model and experimental data, the reduced chi-square (χ^2_{red}) value exceeding 1 implies an under-fitting of the experimental data to the model. This might be attributed to large residuals during the fitting process, particularly for the data in the temperature ranges of 260-310 °C and 310-360 °C (Fig. 4b). The fluctuating trend in measured L (μm) within these temperature ranges suggests the occurrence of more complicated diffusion processes. Our measurements assumed that the analysed areas were continuous and showed one-dimensional diffusion. These assumptions were to eliminate the potential interference from grain boundaries, which introduce additional interfacial energy when chemical elements transport between grains, and to use a one-dimensional thin film solution of Fick's second law in deriving the diffusion model. There may be grain boundaries within the measured areas and the diffusion within these areas deviate somewhat from one-dimensional diffusion.

A close examination of the experimental data reveals the measured L systematically above or below the fitted curve in diffusion length at 310-320 °C and 340-360 °C, implying the occurrence of complicated processes possibly related to grain boundaries or none-one-dimensional diffusion in the measured areas. These complexities might not only increase the residuals between the proposed model and experimental data but also contribute to the large uncertainties in the fitting parameters for the function $L(T)$ (equation S8). The standard errors on the parameters were calculated by taking the square root of the diagonals of the covariance matrix after fitting the data with the curve_fit function. The standard errors (one standard deviation in the errors) for parameters were estimated

as ±46421.4 (56.4%) for D_0 and ±3032.8 (3.4%) for Q. Despite the small standard error for Q, indicating a well-constrained parameter, the large estimated uncertainties for D_0 (~56.4% of the fitted value) implies a lack of constraint. This could be attributed to limited data points, high variance in experimental data, and high residuals between the experimental data and the fitting model. Nevertheless, a residual histogram (Fig. S19) shows a distribution centred around zero for equation S8, indicating that the model effectively captures the trend of the measured data[35]. From a range of data assessments, we believe that the proposed model adequately describes sodium diffusion in continuous oxide layers, provided there are no significant grain boundary effects and the diffusion is approximately one-dimensional. The fitting parameters allow us to derive diffusion coefficients of sodium at different temperatures, as presented in Table S2. These values are in good agreement with those calculated for sodium diffusion in solid oxides using density functional theories (DFT)[36].

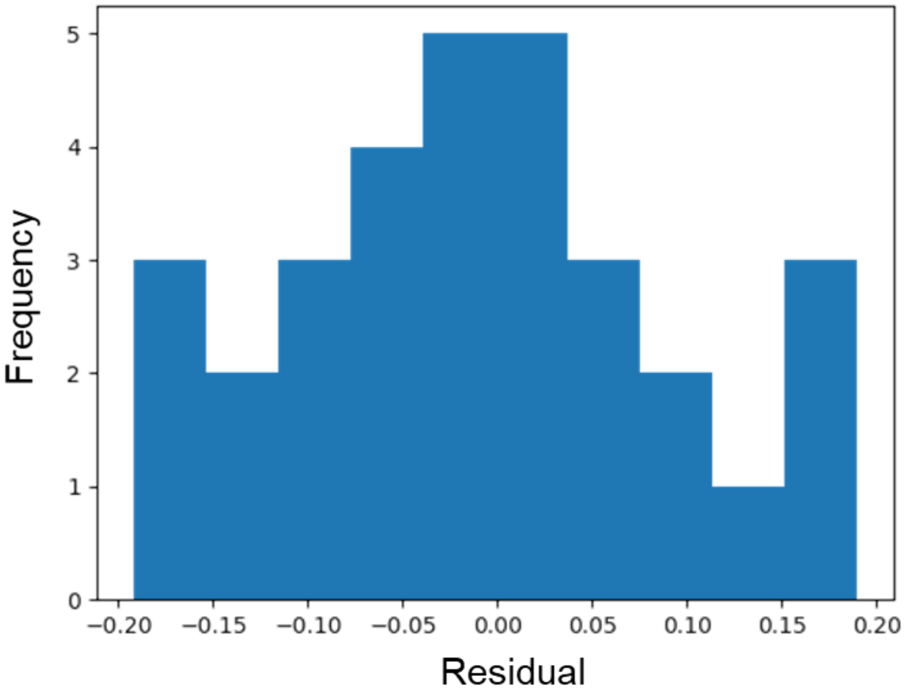

Figure S19. Histograms showing the distribution of the residuals for the proposed model used for the fitting.

Table S2. Solid-state diffusion coefficient of sodium in the oxide layers at individual temperatures.

**Temperature** (℃)	$\boldsymbol{D}$ ($\boldsymbol{T}$) (μm^2/sec)	$\boldsymbol{D}$ ($\boldsymbol{T}$) (cm^2/sec)
261.5	0.0002	2.02E^{-12}
267.14	0.0002	2.48E^{-12}
277.01	0.0004	3.53E^{-12}
286.88	0.0005	4.95E^{-12}
296.75	0.0007	6.88E^{-12}
299.57	0.0008	7.54E^{-12}
306.62	0.0009	9.45E^{-12}
308.03	0.0010	9.88E^{-12}
309.44	0.0010	1.03E^{-11}
315.08	0.0012	1.23E^{-11}
323.54	0.0016	1.59E^{-11}
326.36	0.0017	1.73E^{-11}
332	0.0020	2.03E^{-11}
339.05	0.0025	2.49E^{-11}
343.28	0.0028	2.80E^{-11}
350.33	0.0034	3.41E^{-11}
355.97	0.0040	3.97E^{-11}
360.2	0.0044	4.44E^{-11}
361.61	0.0046	4.61E^{-11}
364.43	0.0050	4.96E^{-11}
368.66	0.0055	5.53E^{-11}
370.07	0.0057	5.74E^{-11}
375.71	0.0066	6.62E^{-11}
379.94	0.0074	7.36E^{-11}
382.76	0.0079	7.89E^{-11}
385.58	0.0085	8.46E^{-11}
389.81	0.0094	9.37E^{-11}
391.22	0.0097	9.70E^{-11}
392.63	0.0100	1.00E^{-10}
394.04	0.0104	1.04E^{-10}
398.27	0.0115	1.15E^{-10}

References

(1) Pham, S. T.; Tieu, A. K.; Wan, S.; Hao, J.; Zhu, H.; Nguyen, H. H.; Mitchell, D. R. G. Oxidative and Frictional Behavior of a Binary Sodium Borate–Silicate Composite in High-Temperature Lubricant Applications. *Ind. Eng. Chem. Res.* **2020**, *59* (7), 2921-2933.

(2) Pham, S. T.; Tieu, A. K.; Wan, S.; Hao, J.; Zhu, H.; Tran, N. V.; Do, P. T. Intrinsic Effect of Alkali Concentration on Oxidation Reactivity and High-Temperature Lubricity of Silicate Melts between Rubbed Steel/Steel Contacts. *Langmuir* **2020**, *36* (27), 7850-7860.

(3) Pham, S. T.; Tieu, A. K.; Sencadas, V.; Nancarrow, M. J. B.; Peleckis, G.; Nguyen, H. H. Insight into the Mechanical Behavior of Hybrid Colloidal Capsules at Elevated Temperatures by Direct Visualization of the Interfacial Solid-State Reactions. *J. Phys. Chem. C .* **2021**, *125* (31), 17462-17473.

(4) Pham, S. T.; Tieu, K. A.; Wan, S.; Lei, W.; Liu, D.; Tran, N. V. Anti-oxidation mechanism and interfacial chemistry of BN@CaCO3-SiO2 microcapsule-added sodium borate melt on the sliding steel surfaces at elevated temperatures. *Appl. Surf. Sci.* **2021**, 150556.

(5) Burnett, T. L.; Kelley, R.; Winiarski, B.; Contreras, L.; Daly, M.; Gholinia, A.; Burke, M. G.; Withers, P. J. Large volume serial section tomography by Xe Plasma FIB dual beam microscopy. *Ultramicroscopy* **2016**, *161*, 119-129.

(6) Ostadi, H.; Jiang, K.; Prewett, P. D. Characterisation of FIB milling yield of metals by SEM stereo imaging technique. *Microelectronic Engineering* **2009**, *86* (4), 1021-1024.

(7) Collette, A.; ; Kluyver, T.; ; Caswell, T. A.; ; Tocknell, J.; ; Kieffer, J.; ; et al. *h5py/h5py: 3.8.0-aarch64-wheels*; (2023). DOI: 10.5281/zenodo.594310.

(8) Peña, F. d. l.; ; Prestat, E.; ; Fauske, V. T.; ; Burdet, P.; ; Lähnemann, J.; ; et al. *hyperspy/hyperspy: Release v1.6.5*; (2022). DOI: 10.5281/zenodo.5608741.

(9) Trindade, G. F.; Abel, M.-L.; Lowe, C.; Tshulu, R.; Watts, J. F. A Time-of-Flight Secondary Ion Mass Spectrometry/Multivariate Analysis (ToF-SIMS/MVA) Approach To Identify Phase Segregation in Blends of Incompatible but Extremely Similar Resins. *Analytical Chemistry* **2018**, *90* (6), 3936-3941.

(10) Collins, S. M.; Kepaptsoglou, D. M.; Hou, J.; Ashling, C. W.; Radtke, G.; Bennett, T. D.; Midgley, P. A.; Ramasse, Q. M. Functional Group Mapping by Electron Beam Vibrational Spectroscopy from Nanoscale Volumes. *Nano. Lett.* **2020**, *20* (2), 1272-1279.

(11) Collins, S. M.; Kepaptsoglou, D. M.; Butler, K. T.; Longley, L.; Bennett, T. D.; Ramasse, Q. M.; Midgley, P. A. Subwavelength Spatially Resolved Coordination Chemistry of Metal–Organic Framework Glass Blends. *J. Am. Chem. Soc.* **2018**, *140* (51), 17862-17866.

(12) Lin, C. Projected Gradient Methods for Nonnegative Matrix Factorization. *Neural Computation* **2007**, *19* (10), 2756-2779.

(13) Berry, M. W.; Browne, M.; Langville, A. N.; Pauca, V. P.; Plemmons, R. J. Algorithms and applications for approximate nonnegative matrix factorization. *Computational Statistics & Data Analysis* **2007**, *52* (1), 155-173.

(14) Smith, N. S.; Skoczylas, W. P.; Kellogg, S. M.; Kinion, D. E.; Tesch, P. P.; Sutherland, O.; Aanesland, A.; Boswell, R. W. High brightness inductively coupled plasma source for high current focused ion beam applications. *Journal of Vacuum Science & Technology B: Microelectronics and Nanometer Structures Processing, Measurement, and Phenomena* **2006**, *24* (6), 2902-2906.

(15) Hou, J.; Chen, P.; Shukla, A.; Krajnc, A.; Wang, T.; Li, X.; Doasa, R.; Tizei, L. H. G.; Chan, B.; Johnstone, D. N.; et al. Liquid-phase sintering of lead halide perovskites and metal-organic framework glasses. *Science* **2021**, *374* (6567), 621-625.

(16) Tran, B. H.; Tieu, A. K.; Wan, S.; Zhu, H.; Mitchell, D. R.; Nancarrow, M. J. Multifunctional Bi-Layered Tribofilm Generated on Steel Contact Interfaces under High-Temperature Melt Lubrication. *J. Phys. Chem. C .* **2017**, *121* (45), 25092-25103.

(17) Pham, S. T.; Huynh, K. K.; Tieu, K. A. Tribological performances of ceramic oxide nanoparticle additives in sodium borate melt under steel/steel sliding contacts at high temperatures. *Tribo. Int.* **2022**, *165*, 107296.

(18) Whitby, J. A.; Östlund, F.; Horvath, P.; Gabureac, M.; Riesterer, J. L.; Utke, I.; Hohl, M.; Sedláček, L.; Jiruše, J.; Friedli, V. High spatial resolution time-of-flight secondary ion mass spectrometry for the masses: a novel orthogonal ToF FIB-SIMS instrument with in situ AFM. *Advances in Materials Science and Engineering* **2012**, *2012*, 180437.

(19) Senoner, M.; Unger, W. Lateral resolution of secondary ion mass spectrometry–results of an inter-laboratory comparison. *Surface and Interface Analysis: An International Journal devoted to the development and application of techniques for the analysis of surfaces, interfaces and thin films* **2007**, *39* (1), 16-25.

(20) Xu, D.; Hua, X.; Liu, S.-C.; Qiao, H.-W.; Yang, H.-G.; Long, Y.-T.; Tian, H. In situ and real-time ToF-SIMS analysis of light-induced chemical changes in perovskite CH3NH3PbI3. *ChemComm.* **2018**, *54* (43), 5434-5437.

(21) Otto, S.-K.; Riegger, L. M.; Fuchs, T.; Kayser, S.; Schweitzer, P.; Burkhardt, S.; Henss, A.; Janek, J. In Situ Investigation of Lithium Metal–Solid Electrolyte Anode Interfaces with ToF-SIMS. *Adv. Mater. Interfaces.* **2022**, *9* (13), 2102387.

(22) Pillatsch, L.; Östlund, F.; Michler, J. FIBSIMS: A review of secondary ion mass spectrometry for analytical dual beam focussed ion beam instruments. *Prog. Cryst. Growth Charact. Mater.* **2019**, *65* (1), 1-19.

(23) Harvey, S. P.; Zhang, F.; Palmstrom, A.; Zhu, K.; Luther, J. M.; Berry, J. Understanding Measurement Artifacts Causing Inherent Cation Gradients in Depth Profiles of Perovskite Photovoltaics with TOF-SIMS. In *2019 IEEE 46th Photovoltaic Specialists Conference (PVSC)*, 16-21 June 2019, 2019; pp 1487-1490. DOI: 10.1109/PVSC40753.2019.8980549.

(24) Belianinov, A.; Burch, M. J.; Hysmith, H. E.; Ievlev, A. V.; Iberi, V.; Susner, M. A.; McGuire, M. A.; Maksymovych, P.; Chyasnavichyus, M.; Jesse, S.; et al. Chemical Changes in Layered Ferroelectric Semiconductors Induced by Helium Ion Beam. *Sci Rep* **2017**, *7* (1), 16619.

(25) Mouhib, T.; Poleunis, C.; Wehbe, N.; Michels, J. J.; Galagan, Y.; Houssiau, L.; Bertrand, P.; Delcorte, A. Molecular depth profiling of organic photovoltaic heterojunction layers by ToF-SIMS: comparative evaluation of three sputtering beams. *Analyst* **2013**, *138* (22), 6801-6810.

(26) Mooney, P. M.; Cheng, L. J.; Süli, M.; Gerson, J. D.; Corbett, J. W. Defect energy levels in boron-doped silicon irradiated with 1-MeV electrons. *Physical Review B* **1977**, *15* (8), 3836-3843.

(27) Bracht, H.; Radek, M.; Kube, R.; Knebel, S.; Posselt, M.; Schmidt, B.; Haller, E. E.; Bougeard, D. Ion-beam mixing in crystalline and amorphous germanium isotope multilayers. *Journal of Applied Physics* **2011**, *110* (9).

(28) Tran, B. H.; Tieu, A. K.; Wan, S.; Zhu, H.; Liu, R. Hot Corrosion of Borate Melt and Interface Chemistry of Borate-Coated Steel Under Tribological Stimulation. *Corros. Sci.* **2018**, *140*, 231-240.

(29) Paul, A.; Laurila, T.; Vuorinen, V.; Divinski, S. V. Fick's Laws of Diffusion. In *Thermodynamics, Diffusion and the Kirkendall Effect in Solids*, Paul, A., Laurila, T., Vuorinen, V., Divinski, S. V. Eds.; Springer International Publishing, 2014; pp 115-139.
(30) Nemouchi, F.; Mangelinck, D.; Bergman, C.; Gas, P.; Smith, U. Differential scanning calorimetry analysis of the linear parabolic growth of nanometric Ni silicide thin films on a Si substrate. *Appl. Phys. Lett.* **2005**, *86* (4), 041903.
(31) Tyurnina, Z. G.; Tyurnina, N. G.; Sviridov, S. I. Diffusion of sodium ions and electric conductivity in glassy and crystallized Na2O · Al2O3 · 2SiO2. *Glass Physics and Chemistry* **2015**, *41* (6), 579-581.
(32) Schoo, U.; Mehrer, H. Diffusion of 22Na in sodium borate glasses. *Solid State Ion.* **2000**, *130* (3), 243-258.
(33) Regev, S.; Grønbech-Jensen, N.; Farago, O. Isothermal Langevin dynamics in systems with power-law spatially dependent friction. *Physical Review E* **2016**, *94* (1), 012116.
(34) Frederiksen, J. M.; Mejlbro, L.; Nilsson, L.-O. *Fick's 2nd law-Complete solutions for chloride ingress into concrete–with focus on time dependent diffusivity and boundary condition. (Report TVBM; Vol. 3146)*; Division of Building Materials, LTH, Lund University, 2009.
(35) Andrae, R.; Schulze-Hartung, T.; Melchior, P. Dos and don'ts of reduced chi-squared. *arXiv preprint arXiv:1012.3754* **2010**.
(36) Jung, S. C.; Kim, H.-J.; Choi, J. W.; Han, Y.-K. Sodium Ion Diffusion in Al2O3: A Distinct Perspective Compared with Lithium Ion Diffusion. *Nano. Lett.* **2014**, *14* (11), 6559-6563.
